# Restoring calcium homeostasis in Purkinje cells arrests neurodegeneration and neuroinflammation in the ARSACS mouse model

**DOI:** 10.1172/jci.insight.163576

**Published:** 2023-06-22

**Authors:** Andrea Del Bondio, Fabiana Longo, Daniele De Ritis, Erica Spirito, Paola Podini, Bernard Brais, Angela Bachi, Angelo Quattrini, Francesca Maltecca

**Affiliations:** 1 Mitochondrial Dysfunctions in Neurodegeneration Unit, Division of Neuroscience, Ospedale San Raffaele, Milan, Italy.; 2 Università Vita-Salute San Raffaele, Milan, Italy.; 3 Experimental Neuropathology Unit, Division of Neuroscience and Institute of Experimental Neurology, Ospedale San Raffaele, Milan, Italy.; 4 Department of Neurology and Neurosurgery, Montreal Neurological Institute and Hospital, McGill University, Montreal, Quebec, Canada.; 5 IFOM- FIRC Institute of Molecular Oncology, Milan, Italy.

**Keywords:** Cell Biology, Neuroscience, Genetic diseases, Neurodegeneration

## Abstract

Autosomal recessive spastic ataxia of Charlevoix-Saguenay (ARSACS) is caused by mutations in *SACS* gene encoding sacsin, a huge protein highly expressed in cerebellar Purkinje cells (PCs). Patients with ARSACS, as well as mouse models, display early degeneration of PCs, but the underlying mechanisms remain unexplored, with no available treatments. In this work, we demonstrated aberrant calcium (Ca^2+^) homeostasis and its impact on PC degeneration in ARSACS. Mechanistically, we found pathological elevation in Ca^2+^-evoked responses in *Sacs^–/–^* PCs as the result of defective mitochondria and ER trafficking to distal dendrites and strong downregulation of key Ca^2+^ buffer proteins. Alteration of cytoskeletal linkers, which we identified as specific sacsin interactors, likely account for faulty organellar trafficking in *Sacs^–/–^* cerebellum. Based on this pathogenetic cascade, we treated *Sacs^–/–^* mice with Ceftriaxone, a repurposed drug that exerts neuroprotection by limiting neuronal glutamatergic stimulation and, thus, Ca^2+^ fluxes into PCs. Ceftriaxone treatment significantly improved motor performances of *Sacs^–/–^* mice, at both pre- and postsymptomatic stages. We correlated this effect to restored Ca^2+^ homeostasis, which arrests PC degeneration and attenuates secondary neuroinflammation. These findings disclose key steps in ARSACS pathogenesis and support further optimization of Ceftriaxone in preclinical and clinical settings for the treatment of patients with ARSACS.

## Graphical abstract

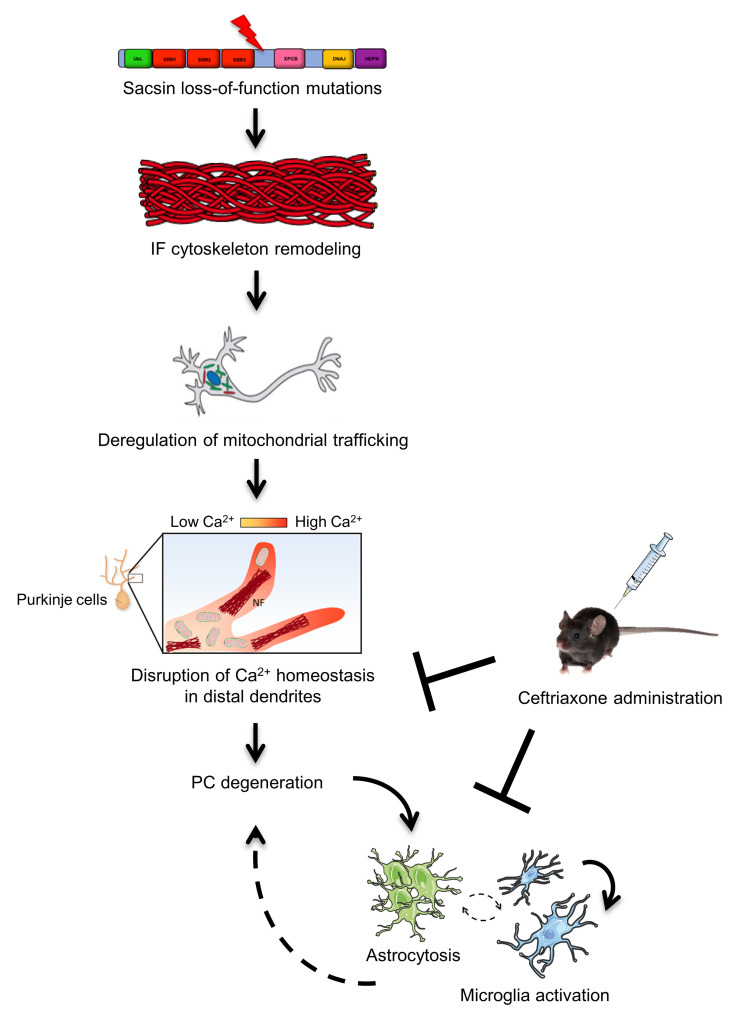

## Introduction

Autosomal recessive spastic ataxia of Charlevoix-Saguenay (ARSACS; MIM #270550) is a childhood-onset neurodegenerative disease characterized by cerebellar ataxia, followed by pyramidal tract signs and peripheral neuropathy (1, 2). ARSACS is caused by loss-of-function mutations in *SACS* gene, with more than 200 mutations identified worldwide (3).

The *SACS* gene encodes the massive 520 kDa protein sacsin, which is mainly expressed in the CNS, with the highest levels in Purkinje cells (PCs) in the cerebellum (4–6). Loss of PCs is indeed the most prominent pathological feature in patients with ARSACS (1) and in mouse models (*Sacs^–/–^* and *Sacs^R272C/R272C^*). These models recapitulate the human phenotype presenting with ataxia in the early phases of disease, while spasticity and peripheral neuropathy occur at later stages (6–8).

Sacsin is a multimodular protein composed of an ubiquitin-like (UbL) domain that binds to the proteasome (5), 3 sacsin repeating regions (SRR) having high homology with Hsp90 (9), an XPCB domain (10), a DnaJ domain that binds Hsc70 (5), and a HEPN domain (11). Despite that the nature of these motifs suggests that sacsin may operate in protein quality control, the cellular function of this protein and the pathophysiological consequences of its dysfunction remain largely unknown.

We previously found that the absence of sacsin affects intermediate filament (IF) organization, with abnormal bundles of vimentin in fibroblasts of patients with ARSACS (12) and of neurofilament (NFs) in brain autopsies of patients with ARSACS and in the *Sacs^–/–^* mouse (8).

Mitochondrial dysfunction was also reported in cell models of ARSACS, with reduced oxygen consumption rates (OCR) and increased reactive oxygen species (ROS) production (13, 14). A more interconnected mitochondrial network was observed in the same cell models and in dissociated dorsal root ganglia (DRG) neurons from the *Sacs^–/–^* mouse (7, 8, 13).

However, if and how these phenotypes are mechanistically interlinked and cause PC degeneration remains unsolved. Published studies were mostly performed in cell models (fibroblasts, HEK, or SH-SY5Y cells) that do not recapitulate the complexity of PC morphology and physiology, or in sacsin-depleted cultured neurons that are not primarily affected in the disease (hippocampal neurons or DRGs; refs. 7, 15). Indeed, cerebellar PCs are highly specialized neurons characterized by unique firing properties, high rate of metabolism, and an extreme cytoarchitecture, with an extensively branched dendritic tree receiving mostly glutamatergic stimulation (16).

Up to now, a detailed study of the mechanisms underlying PC degeneration is lacking, impeding the development of targeted treatments for ARSACS.

In this work, by employing multiple experimental approaches in vivo in the cerebellum and ex vivo in primary PCs from the *Sacs^–/–^* mouse model, we uncovered key aspects of cerebellar degeneration in ARSACS. We demonstrated a strong alteration of Ca^2+^ homeostasis in *Sacs^–/–^* mice. Our results suggest that this phenotype could be secondary to defective mitochondria and ER trafficking to distal dendrites, likely as the result of alterations in specific cytoskeletal linker proteins involved in organellar transport. Moreover, many proteins related to the regulation of Ca^2+^ homeostasis (Ca^2+^ buffers and Ca^2+^ATP-ases) were strongly reduced in *Sacs^–/–^* mice. We also found that Ca^2+^-induced PC degeneration triggers a neuroinflammatory response in the cerebellum, with pronounced astrocytosis and microgliosis, supported by both IHC and RNA-Seq data.

As a proof of concept of this pathogenetic cascade, we show that the post- and presymptomatic administration of Ceftriaxone, a drug able to reduce glutamatergic stimulation and, thus, Ca^2+^ influx in neurons (17, 18), ameliorates motor symptoms and arrests PC degeneration in the *Sacs^–/–^* mouse. We demonstrated that this beneficial effect is likely achieved by restored Ca^2+^ homeostasis in PCs and attenuated neuroinflammation in the cerebellum. Optimized Ceftriaxone treatment might represent a therapeutic perspective for ARSACS.

## Results

### Sacs^–/–^ PCs show defective trafficking of mitochondria and ER.

*Sacs^–/–^* mice display an early accumulation of nonphosphorylated neurofilament heavy polypeptide (npNFH) in the cerebellum at P15 (Supplemental Figure 1A; supplemental material available online with this article; https://doi.org/10.1172/jci.insight.163576DS1). To mechanistically dissect the downstream effects of npNFH accumulation at a cellular level, we employed (a) *SACS^–/–^* SH-SY5Y cells that we previously generated (19) and (b) *Sacs^–/–^* primary cerebellar cultures enriched in PCs. By confocal imaging, we demonstrated a striking IF accumulation in *SACS^–/–^* SH-SY5Y cells, both undifferentiated (vimentin and npNFH) (Supplemental Figure 1B) and differentiated into neurons (npNFH) (Supplemental Figure 1C). We also observed npNFH accumulation in *Sacs^–/–^* PCs in primary cerebellar cultures, that was evident in axon and proximal dendrites at 10 days in vitro (DIV10) (Supplemental Figure 2A) and even more prominent at DIV15, a stage at which PCs are arborized and spiny (Supplemental Figure 2B). At this stage, the morphology of *Sacs^–/–^* and WT PCs was comparable, as supported by unchanged total volume (Supplemental Figure 2C). No major defects in microtubules and microfilaments were observed in *SACS^–/–^* SH-SY5Y cells differentiated into neurons as well as in *Sacs^–/–^* primary PCs (Supplemental Figure 2, D–F), indicating IF remodeling as a primary event in ARSACS pathogenesis.

Since *Sacs^–/–^* primary PCs nicely recapitulate the in vivo ARSACS pathophysiology, we next explored if npNFH accumulation in proximal dendrites may impact organelle trafficking to the periphery.

We first focused on mitochondria, which are essential for ATP supply and Ca^2+^ buffering in distal dendrites of PCs, receiving massive glutamatergic stimulation (18, 20). Long-range transport of mitochondria occurs along microtubules, while actin filaments and NFs mediate short-range movement, docking and transient immobilization (21).

**Figure 1:**
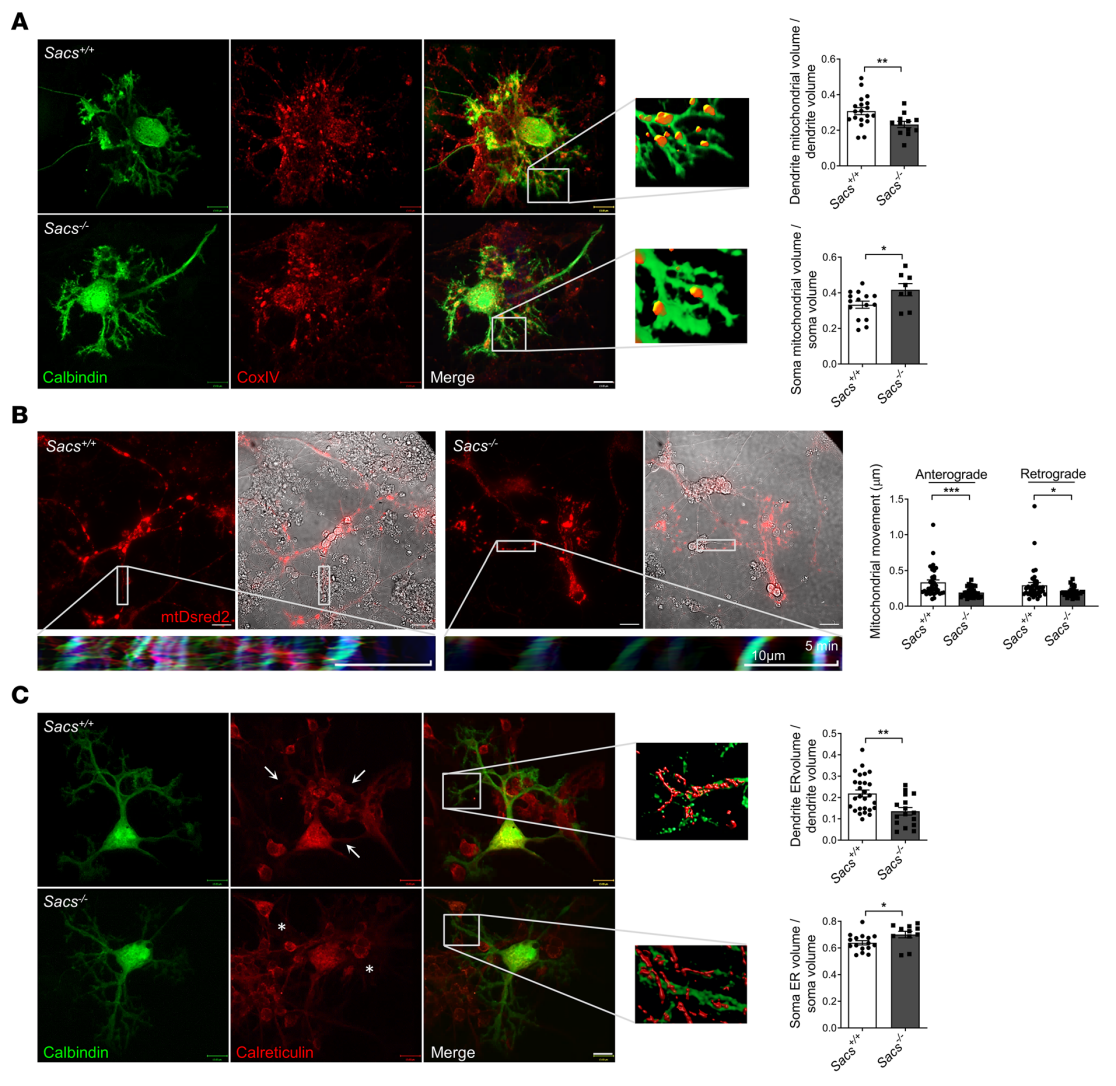
Defective mitochondrial and ER distribution in distal processes of *Sacs^–/–^* primary PCs. (**A**) Representative confocal images (63***×***) and higher-magnification rendering of distal dendrites of *Sacs^–/–^* and WT PCs at DIV15 stained in green with calbindin and in red with the mitochondrial marker CoxIV. Scale bar: 13 μm. Graphs represent volumetric quantification of dendritic or soma mitochondrial volume (normalized to dendrite/soma volume). Data are shown as mean ± SEM; *n* = at least 9 from at least 5 independent experiments; Welch’s *t* test: *P < 0.05, **P < 0.01. (**B**) Representative images (60***×***) of *Sacs^–/–^* and WT PC dendrites at DIV10 after infection with mtDsred2 lentivirus. Scale bar: 20 μm. Kymographs were derived by 30 minutes of live imaging (1 frame/minute) using KymographClear (ImageJ; NIH). The kymographs are relative to the dendrite highlighted into the white boxes (the orientation is with soma to the left). Color code: in red, the anterograde movement; in green, retrograde movement; and in blue, the still mitochondria. Graphs represent the average anterograde or retrograde displacement per minute along dendrite longitudinal axis of each mitochondrion. Data are shown as mean ± SEM; *n* = at least 34 from at least 3 independent experiments; Welch’s *t* test: *P < 0.05, ***P < 0.001. (**C**) Representative confocal images (63***×***) and higher-magnification rendering of distal dendrites of *Sacs^–/–^* and WT PCs at DIV15 stained in green with calbindin and in red with the ER marker calreticulin. Scale bar: 13 μm. Graphs represent volumetric quantification of dendritic or soma ER volume (normalized to dendrite/soma volume). Data are shown as mean ± SEM; *n* = at least 11 from at least 6 independent experiments; Welch’s *t* test: *P < 0.05, **P < 0.01.

By immunofluorescence staining and 3D reconstruction of confocal stack images, we found that mitochondria were retained in the cell soma and not properly distributed in distal dendrites of *Sacs^–/–^* PCs at DIV15. Indeed, quantitative image analysis showed that the volume of mitochondria in PC dendrites was significantly reduced in *Sacs^–/–^* neurons compared with WT controls, while the volume of mitochondria in the soma was increased (Figure 1A). To complement this volumetric analysis, we performed live imaging of mitochondrial movement in PCs by infecting primary cerebellar cultures with mitochondrial recombinant *Discosoma sp.* red fluorescent protein (mtDsred2). PCs were clearly distinguished in bright-field for their peculiar morphology, as they were markedly larger and more ramified than granule cells or inhibitory interneurons. Quantitative analysis showed that both retrograde and anterograde movement rate were significantly reduced in *Sacs^–/–^* versus *Sacs^+/+^* PCs, as well as the total distance traveled by each mitochondrion (Figure 1B, Supplemental Figure 3A, Supplemental Movies 1 and 2). No differences were found in total PC mitochondrial volume (normalized to total PC volume) (Supplemental Figure 3B). Mitochondrial biogenesis was not found to be affected in *Sacs^–/–^* mice compared with *Sacs^+/+^* mice, as demonstrated by unaltered levels of the master regulator of this pathway (PGC1-α, both mRNA and protein; ref. 22) and of its downstream targets *Nrf1* and *Nrf2* (Supplemental Figure 3, C and D).

We demonstrated that cytoskeletal disorganization in the absence of sacsin also impacts ER, which is crucial in PC spines as well as mitochondria in local Ca^2+^ storage and synaptic plasticity (23, 24). Immunofluorescence assay followed by volumetric analysis revealed a reduced amount of ER into dendrites of *Sacs^–/–^* PCs versus WT controls at DIV15. As for mitochondria, the volume of ER in the soma was increased, while no differences were found in total PC ER volume (normalized to total PC volume) (Figure 1C and Supplemental Figure 3E). The thinness of the PC axon prevented us from performing organellar volumetric analysis, as done in dendrites.

To further confirm a failure in organelle transport in vivo in the absence of sacsin, we purified synaptosomal fractions from cerebellum and quantified structural markers of mitochondria (AFG3L2) and ER (calreticulin) by WB. This analysis unraveled a slight but significant reduction of these proteins in synaptosomes derived from *Sacs^–/–^* cerebellum compared with WT controls (Supplemental Figure 3F).

### Mitochondrial ultrastructure and functionality are not altered in Sacs^–/–^ cerebellum.

**Figure 2:**
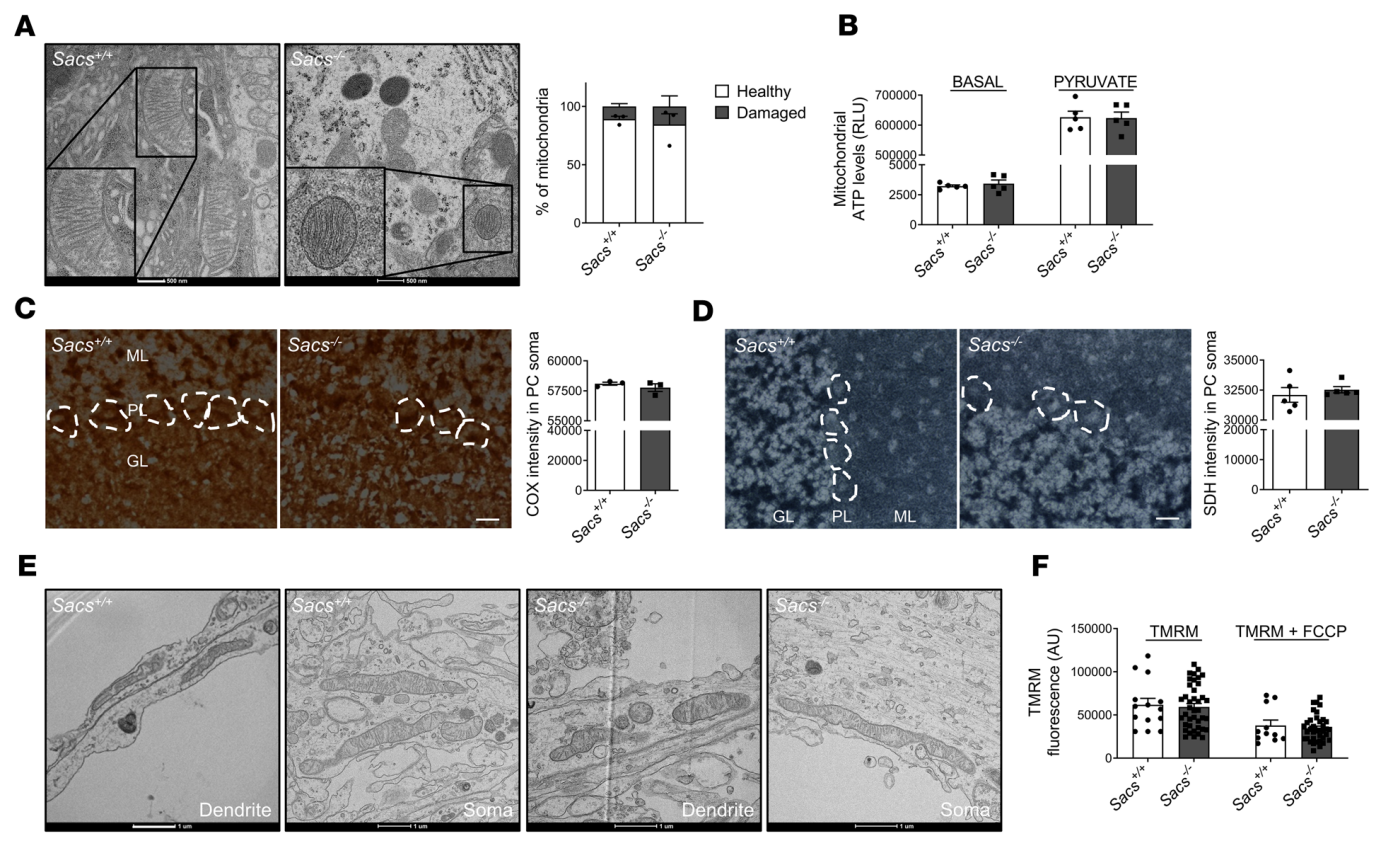
In vivo and ex vivo *Sacs^–/–^* PCs show unaltered mitochondrial ultrastructure and normal mitochondrial metabolism. (**A**) Representative EM images (150,000***×*** magnification) and relative quantitation of PC showing intact inner and outer mitochondrial membrane in *Sacs^–/–^* PCs at 5 months of age. Scale bar: 500 nm. Data are shown as mean ± SEM; *n* = 3; Welch’s *t* test. (**B**) ATP production analysis in freshly isolated mitochondria from mouse cerebellum at 5 months of age at basal level and upon pyruvate stimulation. Data are shown as mean ± SEM; *n* = 5; Welch’s *t* test. (**C** and **D**) Colorimetric activity assay for COX (**C**) and SDH (**D**) in fresh cerebellar slices in PC soma of *Sacs^–/–^* and WT mice at 5 months of age (ML, molecular layer; PL, Purkinje cell layer; GL, granule cell layer). Scale bar: 25 μm. Data are shown as mean ± SEM; *n* = 3 (at least 4 images per sample and at least 4 cells per image); Welch’s *t* test. (**E**) Representative EM images (150,000***×*** magnification) of primary PC showing intact mitochondrial ultrastructure in *Sacs^–/–^* PCs at DIV15 (both in soma and processes). Scale bar: 1 μm. (**F**) Analysis of ΔΨ_mito_ by live-imaging measurement of TMRM fluorescence intensity in DIV15 primary PC soma. Data are shown as mean ± SEM; *n* = at least 14 from at least 4 independent experiments; Welch’s *t* test.

Previous papers showed altered mitochondrial respiration in fibroblasts from patients with ARSACS and in *SACS-*knockdown SH-SY5Y cells (13, 14). We, thus, tested if this defect was conserved in the cerebellum in the absence of sacsin. We performed electron microscopy (EM) analysis of PC soma and synaptic terminals in vivo to visualize mitochondrial ultrastructure. High-resolution images underlined intact inner and outer membranes, with well-defined cristae organization both in WT and in *Sacs^–/–^* PCs at 5 months of age (Figure 2A). Quantitative analysis of several mitochondrial structure parameters revealed no differences between *Sacs^–/–^* and WT cerebellum (Figure 2A and Supplemental Figure 3G). We also tested mitochondrial ATP production in freshly isolated mitochondria from cerebellum of *Sacs^–/–^* mice and relative WT controls at the same age. We found no differences in mitochondrial ATP levels produced by *Sacs^–/–^* mice compared with WT, both at basal state and upon stimulation with pyruvate (Figure 2B). To exclude that this result could reflect a dilution effect of PCs in total cerebellum, we assayed respiratory chain functionality by COX (Figure 2C) and SDH (Figure 2D) enzymatic assays in situ on cerebellar cryostat sections; however, this revealed no differences in *Sacs^–/–^* PCs compared with WT samples.

To further strengthen these results, we evaluated mitochondrial ultrastructure and functionality in *Sacs^–/–^* primary PCs. Ultrastructural EM analysis revealed intact morphology and cristae, in both soma and dendrites, in agreement with in vivo data (Figure 2E and quantitative analysis is reported in Supplemental Figure 3H). We then tested mitochondrial membrane potential (ΔΨmito) by live-imaging measurement of the potentiometric dye Tetramethylrhodamine (TMRM), and this revealed no changes in *Sacs^–/–^* PCs versus WT controls at DIV15 (Figure 2F).

Altogether, these results suggest that, although inefficiently trafficked, mitochondria are metabolically unaltered in *Sacs^–/–^* primary PCs as well as in vivo at 5 months, a stage in which motor defects are already manifested in the ARSACS mouse model.

### Alteration of cytoskeletal linkers mediating organellar transport in the absence of sacsin.

To try to find a mechanistic link between sacsin, NF accumulation, and impaired organellar trafficking, we immunoprecipitated endogenous sacsin to identify its interactors. Since both cerebellum and primary cerebellar cultures are heterogeneous in terms of cell populations, with several nonneuronal components, we employed SH-SY5Y cells differentiated into neurons, which express considerable levels of sacsin and that recapitulate npNFH accumulation.

**Figure 3:**
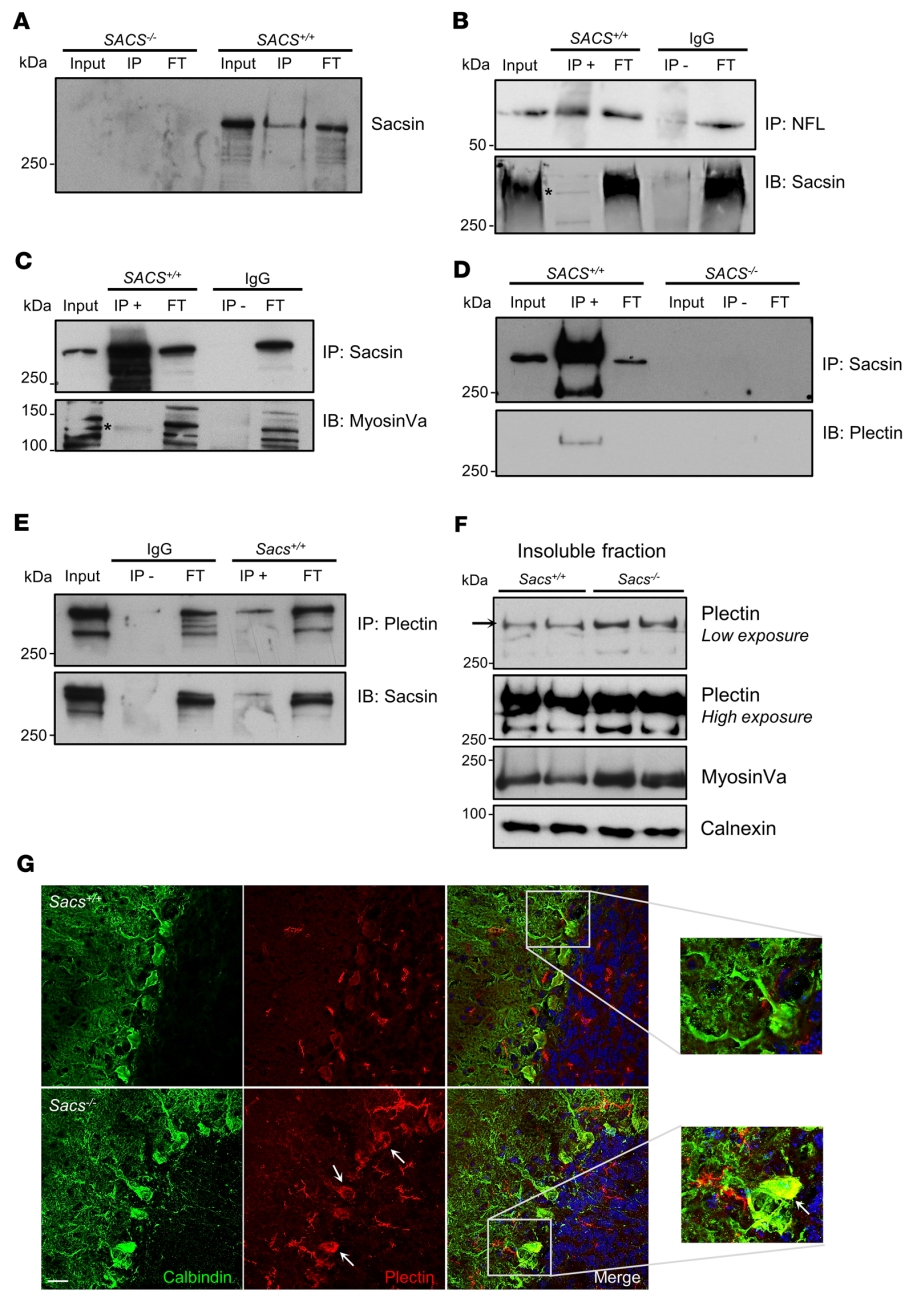
Sacsin physically interacts with plectin and myosin Va, whose levels are deregulated in sacsin-depleted cells and cerebellum. (**A**) Sacsin IP in SH-SY5Y cells (FT, flow through). (**B**) NFL IP in SH-SY5Y cells (with IgG as control) and immunodecoration with anti-sacsin antibody (asterisk indicates the specific band). (**C** and **D**) Sacsin IP in SH-SY5Y cells (with IgG and *SACS^–/–^*, respectively, as control) and immunodecoration with anti–myosin Va (asterisk indicates the specific band )(**C**) and anti-plectin (**D**) antibodies. (**E**) Plectin IP in WT cerebellum (with IgG as control) and immunodecoration with anti-sacsin antibody. (**F**) WB analysis showing levels of plectin and myosin Va in insoluble fractions of *Sacs^–/–^* and WT cerebellum (normalized to calnexin). (**G**) Representative images (60***×***) of cerebellar slices stained with calbindin (in green) and plectin (in red) showing a more intense plectin signal in *Sacs^–/–^* PC soma and proximal dendrites compared with WT controls. Scale bar: 20 μm.

IP of sacsin in WT SH-SY5Y cells differentiated into neurons, followed by label-free quantitative mass spectrometry (LFQ-MS) of eluates, identified 67 specific sacsin interactors (absent in *SACS^–/–^* SH-SY5Y cells used as negative controls, where IP was performed using the same anti-sacsin antibody) (Figure 3A and Supplemental Table 1). STRING network and enrichment analysis underlined that sacsin physical interactors cluster in specific categories related to supramolecular fiber, actin filament and cytoskeleton organization, and organelle localization (Supplemental Figure 4A), supporting the hypothesis that sacsin may exert quality control on cytoskeletal proteins that are crucial for trafficking in highly polarized cells like neurons. NFL and NFM subunits were found directly interacting with sacsin (Table 1), whereas no resident mitochondrial proteins were identified, suggesting that sacsin does not directly interact with mitochondria. Interestingly, among sacsin interactors, we found plectin, a 534 kDa multifunctional cytolinker protein that connects IF with other cytoskeletal components and mitochondria (25, 26), and myosin Va, a 215 kDa protein crucial for both mitochondrial and ER transport in PC dendrites (24) (Table 1). Based on our imaging studies revealing NF bundles and defective organellar trafficking in PCs without gross defects in microtubules and microfilaments, we decided to focus on these interactors. We reconfirmed the interaction between NFL and sacsin, myosin Va and sacsin in SH-SY5Y cells (Figure 3, B and C), and between sacsin and plectin in both SH-SY5Y cells and in the cerebellum (Figure 3, D and E). In support to a potential plectin involvement in ARSACS pathogenesis, we found reduced plectin levels in soluble fractions obtained from a panel of fibroblasts from patients with ARSACS harboring different *SACS* mutations (Supplemental Figure 4B), in 2 different clones of *SACS^–/–^* SH-SY5Y cells (Supplemental Figure 4C), and in *Sacs^–/–^* cerebellum (Supplemental Figure 4D). According to our data, plectin was not reduced per se; rather, it was redistributed in the insoluble-cytoskeletal fraction. Indeed, the WB on total homogenates from the cells of patients with ARSACS, by lysis of whole cellular pellets in Laemmli buffer 2***×*** and loading on mixed acrylamide-agarose gels (that allows to detect putative aggregates up to 600 kDa complexes; ref. 27) show that plectin itself increased drastically in total homogenates of patient cells compared with controls, as well as vimentin (Supplemental Figure 4E). Similarly, the absence of sacsin impacts on plectin and myosin Va (which is neuronally expressed) solubility in vivo, as both proteins increase in the Triton X-100 insoluble fractions from *Sacs^–/–^* cerebellum compared with WT littermates (Figure 3F). To support these biochemical data, we performed plectin immunofluorescence on murine cerebellar sections at 5 months of age. Plectin showed a more intense signal in the soma and proximal dendrites of *Sacs^–/–^* PCs compared with WT samples, in overlap with the NF bundles (Figure 3G and Supplemental Figure 5A). Interestingly, npNFH or plectin immunofluorescence combined with sacsin staining in primary PCs revealed an overlap of the 2 signals (Supplemental Figure 5, B and C). Altogether, these results indicate that, in the absence of sacsin, there is a striking remodeling of cytoskeletal proteins involved in organellar movement that could lead to a failure in global intracellular trafficking.

### Sacs^–/–^ primary PCs show defective cytosolic Ca^2+^ handling.

A fine regulation of free cytosolic Ca^2+^ concentration is crucial in PCs, which receive massive Ca^2+^ influxes in postsynaptic dendrites due to glutamatergic stimulation.

Postsynaptically, Ca^2+^ signals are shaped by cooperation of Ca^2+^-binding proteins, ER, and mitochondria (28). Mitochondria accumulate Ca^2+^ into the matrix themselves but also fuel Ca^2+^ clearance systems — i.e., Ca^2+^ ATP-ases in the plasma membrane and ER (29).

**Figure 4:**
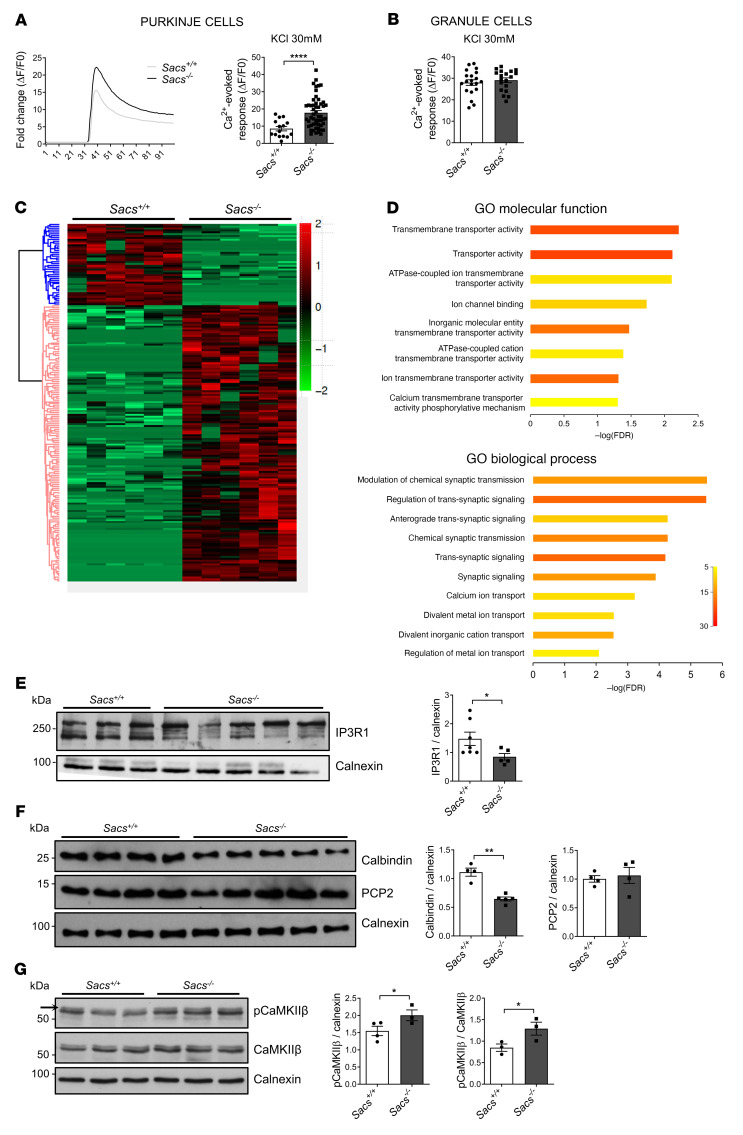
Ca^2+^ deregulation in *Sacs^–/–^* primary PCs and cerebellum. (**A**) Representative traces of cytosolic Ca^2+^ responses before and after KCl stimulation of *Sacs^–/–^* and WT control primary PCs. Graph shows PC Ca^2+^-evoked responses after stimulation with 30 mM KCl (normalized increase measured above the initial value). Data are shown as mean ± SEM; *n* = at least 15 from at least 5 independent experiments; Welch’s *t* test. ****P <0,0001. (**B**) Granule cell Ca^2+^-evoked responses after stimulation with 30 mM KCl (normalized increase measured above the initial value). Data are shown as mean ± SEM; *n* = at least 19 from at least 5 independent experiments; Welch’s *t* test. (**C**) Heatmap of cerebellar protein profile comparing *Sacs^–/–^* and WT controls at 5 months of age; *n* = 6 from 3 biological replicates. (**D**) g:Profiler enrichment of deregulated proteins comparing 5-month-old *Sacs^–/–^* and WT cerebellum, showing the top 10 categories for each GO: molecular function and biological process. Color bar represents number of proteins. (**E**–**G**) WB analysis showing levels of IP3R1 (**E**), Calbindin and PCP2 (**F**), and pCaMKIIβ (upper band as indicated by the arrow) and CaMKIIβ (**G**) in *Sacs^–/–^* and WT control cerebellum at 5 months of age with relative quantitation (normalized to calnexin). Data are shown as mean ± SEM; *n* = at least 4; Welch’s *t* test. *P < 0.05, **P < 0.01.

Given the reduced presence of mitochondria and ER in terminal dendrites of *Sacs^–/–^* PCs, we performed live Ca^2+^ imaging by using the highly sensitive Ca^2+^ probe Calbryte 520 in primary cerebellar cultures at DIV15. Upon challenge with 30 mM KCl, which promotes Ca^2+^ entry by plasma membrane and empties Ca^2+^ stores, we found that the Ca^2+^-evoked peaks (ΔF/F_0_) were significantly increased in *Sacs*^–/–^ PCs compared with WT (Figure 4A), reflecting a defective capacity of *Sacs*^–/–^ PCs to handle Ca^2+^ influxes. This was specific to PC, as Ca^2+^-evoked responses in *Sacs*^–/–^ granule cells in the same cerebellar cultures were comparable with WT (Figure 4B).

### Integrated omics approaches disclose deregulation of Ca^2+^ homeostasis in Sacs^–/–^ cerebellum.

To further explore the potential involvement of Ca^2+^ deregulation in ARSACS pathogenesis, we applied 2 omics approaches in vivo. Firstly, by performing LFQ proteomics on cerebellum at 5 months of age (postsymptomatic stage), we identified 194 proteins that were differentially expressed in *Sacs^–/–^* mice versus controls (44 upregulated and 150 downregulated) (Figure 4C and Supplemental Table 2). Gene ontology (GO) enrichment analyses revealed the deregulation of many proteins related to ion transmembrane transport and, in particular, to Ca^2+^ transport, synaptic transmission, and signaling (Figure 4D). Most proteins belonging to microfilaments and microtubules were unaltered, while we found a strong downregulation of proteins related to Ca^2+^ homeostasis, such as Inositol 1,4,5-trisphosphate receptor type 1 (IP3R1, also validated by WB; Figure 4E), Ca^2+^ ATP-ases (ATP2B2, ATP2A3, ATP2A2), and Ca^2+^-binding proteins (calbindin, also validated by WB; Figure 4F). This reduction is specific and not due to PC loss, as a specific marker of PCs (PCP2, also validated by Western blotting [WB]; Figure 4F) remained unaltered (Supplemental Table 2). The increase of Ca^2+^ concentration is known to promote Ca^2+^ calmodulin–dependent protein kinase type II subunit β (CaMKIIβ) autophosphorylation (30). We thus tested the phosphorylation state of CaMKIIβ, which was drastically increased in *Sacs^–/–^* samples in comparison with WT controls, despite unchanged amount of CaMKIIβ (Figure 4G), further enforcing a deregulation of Ca^2+^ in *Sacs^–/–^* cerebellum.

**Figure 5:**
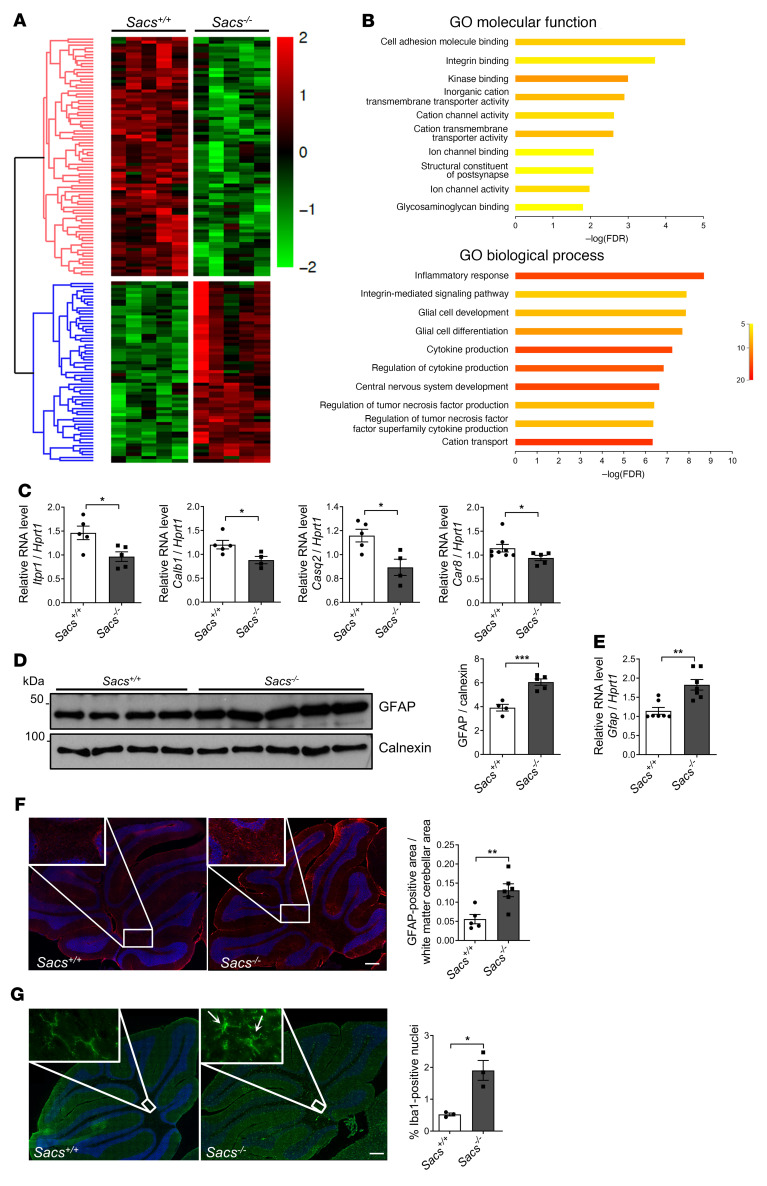
Transcriptomics analysis supports Ca^2+^ deregulation and neuroinflammation in *Sacs^–/–^* cerebellum. (**A**) Heatmap of cerebellar gene expression profile comparing *Sacs^–/–^* and WT mice at 5 months of age; *n* = 5. (**B**) g:Profiler enrichment of deregulated genes comparing 5-month-old *Sacs^–/–^* and WT cerebellum showing the top 10 categories for each GO: molecular function and biological process. Color bar represents number of genes. (**C**) qPCR showing levels of *Itpr1*, *Calb1*, *Casq2*, and *Car8* mRNA (relative to *Hprt1* mRNA) in *Sacs^–/–^* and WT cerebellum at 5 months of age. Data are shown as mean ± SEM; *n* = 5; Welch’s *t* test. * *P* < 0.05. (**D**) WB analysis showing levels of GFAP in *Sacs^–/–^* and WT control cerebellum at 5 months of age with relative quantitation (normalized to calnexin). Data are shown as mean ± SEM; *n* = at least 4; Welch’s *t* test. ***P < 0.001. (**E**) qPCR showing levels of *Gfap* mRNA (relative to *Hprt1* mRNA) in *Sacs^–/–^* and control cerebellum at 5 months of age. Data are shown as mean ± SEM; *n* = 5; Welch’s *t* test. **P < 0.01. (**F**) Representative images of immunofluorescence analysis showing astrocyte activation (GFAP, in red) in 5-month-old *Sacs^–/–^* cerebellum compared with controls. Scale bar: 0.2 mm. Data are shown as mean ± SEM; *n* = 3; Welch’s *t* test. **P < 0.01. (**G**) Representative images of immunofluorescence staining of microglia by Iba1 (in green) highlighting microglial morphological shift in 5-month-old *Sacs^–/–^* cerebellum compared with WT controls. Arrows indicate the amoeboid-phagocytic phenotype of microglia. Quantitative analysis of the percentage of Iba1^+^ cells was normalized to the total nuclei number. Scale bar: 0.2 mm. Data are shown as mean ± SEM; *n* = 3; Welch’s *t* test. *P < 0.05.

We then performed RNA-Seq analysis on *Sacs^–/–^* cerebellar bulk RNA extracts compared with age-matched WT controls at 5 months of age. This analysis revealed 137 deregulated genes in *Sacs^–/–^* cerebellum (59 upregulated and 78 downregulated) (Figure 5A and Supplemental Table 3). GO analysis highlighted that most downregulated genes in the absence of sacsin belong to ion channel transport and activity (GO Molecular Function [GO:MF]) and cation transport (GO Biological Process [GO:BP]), further highlighting a specific Ca^2+^ deregulation (Figure 5B). Interestingly, *Itpr1* and *Calb1* transcripts were downregulated, in agreement with their reduced protein levels. In addition, we found reduction of other transcripts encoding Ca^2+^-related proteins, such as *Casq2*, *Car8*, and *Trpc3* (Figure 5C and Supplemental Table 3).

### PC degeneration in Sacs^–/–^ cerebellum is associated to neuroinflammation.

GO analysis of RNA-Seq data revealed that the most upregulated genes in *Sacs^–/–^* cerebellum were involved in inflammatory response and cytokine production (GO: BP) (Figure 5, A and B), indicating a neuroinflammatory process accompanying PC death.

Consistent with the activation of reactive astrogliosis, GFAP was increased at both protein level (measured by WB and LFQ proteomics) and mRNA level (measured by quantitative PCR [qPCR] and RNA-Seq) at 5 months of age (Figure 5, D and E, and Supplemental Tables 2 and 3). Immunofluorescence staining documented an increased GFAP signal in *Sacs^–/–^* cerebellum compared with WT, especially in the most internal part of cerebellum where the majority of glial cells resides (Figure 5F) (31). These results unlikely reflect a direct role of sacsin on astrocytic IF (GFAP), as indicated by unchanged GFAP levels at 1 month in *Sacs^–/–^* cerebellum (Supplemental Figure 6, A and B) and by unaltered GFAP staining in astrocytes in *Sacs^–/–^* primary cerebellar cultures versus WT (Supplemental Figure 6C).

In addition, many genes typical of the phagocytic microglia (*Itgax*, *Clec7a*, *Cd68*, *Trem2*, *Lpl,* *Pycard*, *Tyrobp*) resulted in strong upregulation by RNA-Seq analysis (Supplemental Table 3). These findings were complemented by immunostaining for the microglial marker Iba1, which revealed a drastic proliferation of microglia and a morphological shift of the microglia toward an amoeboid-phagocytic phenotype in *Sacs^–/–^* slices compared with the homeostatic phenotype of the WT (Figure 5G).

Interestingly, the most upregulated gene in *Sacs^–/–^* cerebellum was *Lcn2* (Supplemental Table 3) encoding for Lipocalin-2, a multifunctional protein synthesized and secreted as an inducible factor from activated microglia, reactive astrocytes, neurons, and endothelial cells in response to brain insults. Several components of the complement system (*C3*, *C3ar1*, *C4b*, *C1qa*, *C1qb*, and *C1qc*), part of brain-innate immune system, were also found strikingly upregulated (Supplemental Table 3).

Altogether, these data support the activation of a neuroinflammatory response that accompanies PC degeneration in *Sacs^–/–^* cerebellum.

### Ceftriaxone administration in Sacs^–/–^ mice ameliorates motor ability and delays PC loss by improving Ca^2+^ homeostasis and neuroinflammation.

Ceftriaxone is a β-lactam antibiotic able to efficiently pass the blood-brain barrier, which is used clinically to treat certain pediatric meningitis. There is robust evidence that Ceftriaxone exerts neuroprotection in many preclinical models of neurodegeneration acting by multiple mechanisms (32, 33). Several studies have documented Ceftriaxone’s ability to reduce glutamate concentration at intersynaptic space and, consequently, Ca^2+^ levels postsynaptically (17), including PCs (18, 34). This evidence provided us a strong rationale to test Ceftriaxone in the ARSACS preclinical model, given the deregulation of Ca^2+^ homeostasis that we documented.

**Figure 6:**
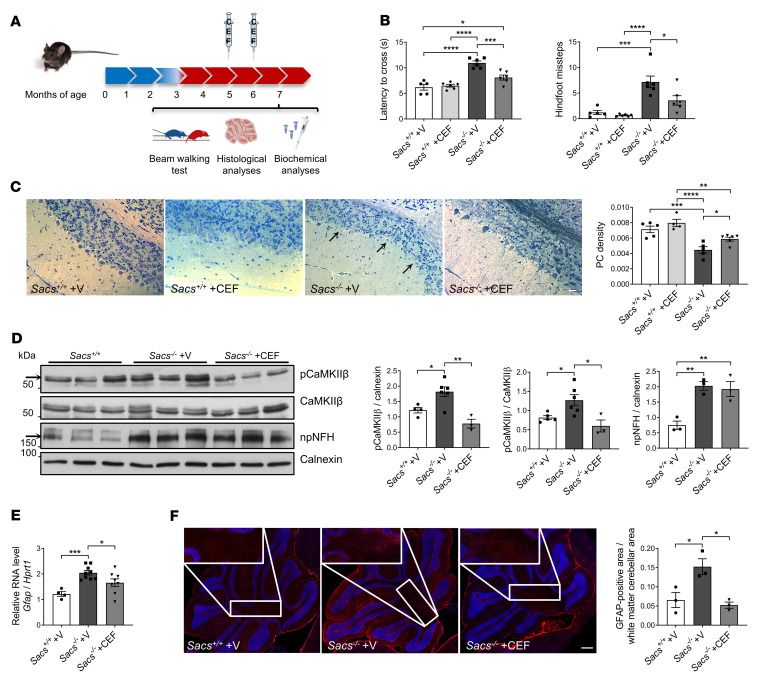
Postsymptomatic Ceftriaxone treatment improves motor coordination, delays PC loss, and mitigates Ca^2+^ deregulation in *Sacs^–/–^* cerebellum. (**A**) Schematic representation of preclinical postsymptomatic Ceftriaxone administration protocol. (**B**) BW test performance in term of latency time to cross the beam and number of hindfoot missteps of female mice of the indicated genotypes, as well as mice that were Ceftriaxone treated and vehicle treated, at 7 months of age. Data are shown as mean ± SEM; *n* = at least 5; 2-way ANOVA with Tukey’s correction. *P < 0.05, ***P < 0.001, ****P < 0.0001. (**C**) Representative semithin sections of cerebellum of Ceftriaxone- and vehicle-treated mice of the indicated genotype, with relative quantitation of PC density at 7 months. Scale bar: 25 μm. Data are shown as mean ± SEM; *n* = at least 4 (10 images per sample); 2-way ANOVA with Tukey’s correction. *P < 0.05, **P < 0.01, ***P < 0.001, ****P < 0.0001. Arrows indicate the PC layer. (**D**) WB analysis showing levels of pCaMKIIβ (upper band as indicated by the arrow), CaMKIIβ, and npNFH in WT, vehicle-treated, and Ceftriaxone-treated *Sacs^–/–^* cerebellum at 7 months of age with relative quantitation (normalized to calnexin). Data are shown as mean ± SEM; *n* = at least 3; 2-way ANOVA with Tukey’s correction. *P < 0.05, **P < 0.01. (**E**) qPCR showing levels of *Gfap* mRNA (relative to *Hprt1* mRNA) in WT, vehicle-treated, and Ceftriaxone-treated *Sacs^–/–^* cerebellum at 7 months of age. Data are shown as mean ± SEM; *n* = at least 4; 2-way ANOVA with Tukey’s correction. *P < 0.05, ***P < 0.001. (**F**) Representative images of immunofluorescence analysis showing astrocytes (GFAP, in red) in 7-month-old Ceftriaxone- and vehicle-treated *Sacs^–/–^* and WT control cerebellum. Data are shown as mean ± SEM; *n* = 3; 2-way ANOVA with Tukey’s correction. *P < 0.05. Scale bar: 0.2 mm.

Since most diagnosed patients with ARSACS are already symptomatic at the time of diagnosis, we first designed a postsymptomatic trial in *Sacs^–/–^* mice. We administered the drug starting at 5 months of age, a stage at which PC loss and motor symptoms are already evident (8). Mice were treated by i.p. injection with 200 mg/kg of Ceftriaxone for 5 consecutive days. In this trial, only 2 cycles of administration (5 and 6 months) were performed (Figure 6A). Before drug administration, we assessed motor incoordination and balance deficit of *Sacs^–/–^* mice by a beam walking (BW) test, which confirmed reduced performances of *Sacs^–/–^* females and males in terms of latency to cross the beam and hindfoot missteps compared with age- and sex-matched WT controls, as previously described (8) (Supplemental Figure 7A). At 7 months, we observed a clear progression of the disease in *Sacs^–/–^* mice treated with vehicle (when compared with their performances at 5 months old mice), which was instead curbed in *Sacs^–/–^* mice treated with Ceftriaxone (Supplemental Movies 3–5). The latency time to cross the beam and the number of hindlimb missteps were remarkably reduced in *Sacs^–/–^*-treated females (Figure 6B) and males (Supplemental Figure 7B) compared with vehicle-treated mice. Semithin sections of anterior lobules of *Sacs^–/–^* Ceftriaxone-treated mice underlined an attenuation of PC degeneration when compared with vehicle-treated *Sacs^–/–^* controls (Figure 6C). By WB analyses of cerebellar extracts, we found that Ceftriaxone treatment strikingly reduced the pathological hyperphosphorylation of CaMKIIβ in *Sacs^–/–^* mice (Figure 6D). We also observed that the levels of the Ca^2+^ buffers calbindin and parvalbumin, and of IP3R1, were increased by Ceftriaxone treatment (Supplemental Figure 7, C and D). These results support that Ceftriaxone ameliorates the motor phenotype and delays PC degeneration in *Sacs^–/–^* mice by acting on cytosolic Ca^2+^ homeostasis, while it is not effective on the accumulation of npNFH (Figure 6D). Moreover, we show evidence supporting that Ceftriaxone administration attenuates astrogliosis, as showed by decreased *Gfap* mRNA (Figure 6E) as well as GFAP^+^ signal in the white matter of the cerebellum (Figure 6F) in Ceftriaxone-treated *Sacs^–/–^* cerebellum compared with vehicle-treated controls.

**Figure 7:**
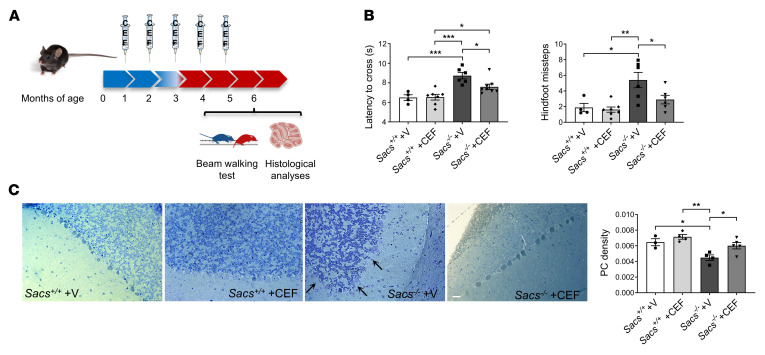
Presymptomatic Ceftriaxone treatment improves motor coordination and delays PC loss in *Sacs^–/–^* mice. (**A**) Schematic representation of preclinical presymptomatic Ceftriaxone administration protocol. (**B**) BW test performance in term of latency time to cross the beam and number of hindfoot missteps at 6 months of age female Ceftriaxone- and vehicle-treated mice. Data are shown as mean ± SEM; *n* = at least 5; 2-way ANOVA with Tukey’s correction. *P < 0.05, **P < 0.01, ***P < 0.001. (**C**) Representative semithin section of 6-month-old cerebellum of Ceftriaxone- and vehicle-treated mice with relative quantitation of PC density. Scale bar: 25 μm. Data are shown as mean ± SEM; *n* = at least 4 (10 images per sample); 2-way ANOVA with Tukey’s correction. *P < 0.05, **P < 0.01.

We also tested Ceftriaxone efficacy at presymptomatic stages, before the onset of disease symptoms. In this preclinical trial, the administration protocol consisted of the same regimen as in the postsymptomatic trial, but the drug was administered starting at 1 month until 5 months of age (5 cycles of administration) (Figure 7A). At 6 months of age, motor assessment by BW test showed an evident improvement of *Sacs^–/–^* Ceftriaxone-treated mice both in latency time to cross the beam and in number of hindlimb missteps compared with placebo-treated age- and sex-matched controls (Figure 7B and Supplemental Figure 8A). Moreover, semithin section analysis demonstrated a reduced PC loss in *Sacs^–/–^* Ceftriaxone-treated cerebellar (anterior lobules) versus vehicle-treated animals (Figure 7C). During both trials, we monitored body weight of treated and untreated mice, and we found no significant differences between the 2 groups (Supplemental Figure 8, B and C). In the presymptomatic trial, the results of hematocrit did not disclose any altered parameter in treated mice. No clear toxicity was observed based on normal values for blood urea, creatinine, albumin, and transaminases ALT parameters, together supporting that repeated Ceftriaxone treatment did not impact kidney or liver function (Supplemental Table 4). In conclusion, Ceftriaxone administration in the ARSACS mouse model with the adopted 5-days-a-month posology appears effective and safe.

## Discussion

In this work, we shed light on the molecular mechanisms underlying PC death in ARSACS and provided evidence of the efficacy of Ceftriaxone in the *Sacs^–/–^* mouse model. The combination of complementary experimental strategies and different models (in vitro, ex vivo, and in vivo), integrated with omics approaches, allowed us to better dissect the cascade of events downstream to the absence of sacsin that leads to PC degeneration.

Although it is well established that loss of sacsin causes IF cytoskeleton derangement in different cell types (6, 12, 15), how this phenotype causes PC degeneration is still unclear. The accumulation of npNFH has been observed in different neurons, but only PCs were found to degenerate in the *Sacs^–/–^* mouse model (8), highlighting a selective susceptibility of these cells in ARSACS. Our data show an early and striking npNFH accumulation in proximal dendrites and axons of *Sacs^–/–^* primary PCs, making these cultures ideal for examining the downstream consequences of cytoskeletal remodeling. In these neurons, we show evidence supporting defective mitochondria and ER trafficking to distal dendrites and Ca^2+^ deregulation in these sites, which is likely to cause PC degeneration in ARSACS.

Previous studies in cell models showed alteration of oxidative phosphorylation and mitochondrial morphology in the absence of sacsin (7, 13, 14). Our ex vivo and in vivo data in *Sacs^–/–^* PCs indicate that mitochondrial morphology was comparable between *Sacs^–/–^* mice and controls, with no signs of hyperfusion or ultrastructural alterations. Accordingly, mitochondrial ATP production was unaltered in *Sacs^–/–^* total cerebellum, as well as COX and SDH activity measured specifically in PCs at 5 months of age, when neurodegeneration is overt.

Although mitochondrial transport occurs mostly on microtubules and actin filaments, there is growing evidence that IF contribute to mitochondrial localization in different cell types, including neurons (35). Transgenic mouse models in which NFs are perturbed show aberrant mitochondrial motility (36, 37). Moreover, extensive dephosphorylation of NFH subunits affects their interaction with mitochondria, leading to a reduced rate of mitochondrial motility (38). These pieces of evidence further support our hypothesis that npNFH accumulation in proximal dendrites of *Sacs^–/–^* PCs could conceivably restrict mitochondrial movement. By live imaging analysis of mitochondrial movement in primary PCs, we indeed found a reduction in both the anterograde and retrograde transport of mitochondria in *Sacs^–/–^* mice versus controls. This is in agreement with a recent paper reporting altered lysosomal positioning in ARSACS fibroblasts and *Sacs^–/–^* mouse cortical neurons (39), that the authors correlate to alteration in microtubule dynamics. Although we could not appreciate gross defects in microtubules in *SACS^–/–^* SH-SY5Y cells as well as ex vivo in *Sacs^–/–^* PCs, and LFQ proteomics in *Sacs^–/–^* cerebellum did not reveal any change in microtubule-related proteins, we cannot exclude that other molecular alterations in microtubules can occur as a consequence of NF bundling.

While the displacement of mitochondria and other organelles to the periphery of vimentin bundles in ARSACS fibroblasts (12) is consistent with our hypothesis of a primary and direct role of sacsin in regulating IF cytoskeleton, the altered mitochondrial functionality observed in ARSACS cell models (7, 13, 14) may rather reflect a secondary effect highlighted by culture conditions that could be milder or more difficult to appreciate in vivo in the *Sacs^–/–^* mouse. Furthermore, most of the specific sacsin interactors that we identified by IP experiments were large cytoskeletal proteins, including NFL and NFM, while no mitochondrial proteins were detected.

In addition to NFs, plectin and myosin Va were found to directly interact with sacsin. Plectin is a giant multifunctional protein acting as a mechanical linker between IF network and other cytoskeletal structures as well as mitochondria (25). Plectin deficiency was demonstrated to affect mitochondrial shape and mostly its transport (26, 40). Class V myosins are actin-based motors that mediate the proper short-range intracellular transport of diverse organelles, mRNAs, and proteins (41). Myosin Va is expressed at high levels in PCs (23), and its deficiency in PCs showed drastic organelle localization defects, as demonstrated by ER missing specifically from dendritic spines (24). Our data showing alterations in mitochondria and ER distribution in distal dendrites in *Sacs^–/–^* primary PCs phenocopy plectin or myosin Va deficiency in neurons, supporting the potential involvement of these 2 proteins in ARSACS pathogenesis (24, 26). We also uncovered accumulation of plectin in cerebellar insoluble fractions from *Sacs^–/–^* mouse cerebellum and, consistently, increased plectin signal in somato-dendritic regions of *Sacs^–/–^* PCs, overlapping the NF bundles. Whether this could reflect a direct role of sacsin on protein quality control of plectin, as well as NFs, needs to be clarified. Further biochemical and functional studies are required to elucidate this point, though it is difficult to address it endogenously in vivo, considering the enormous sizes and stickiness of these cytoskeletal proteins and the possible transient nature of these interactions.

We provided several lines of evidence that Ca^2+^ homeostasis is altered in *Sacs^–/–^* PCs. The control of Ca^2+^ homeostasis is crucial in PCs, since it regulates autonomous pacemaking, as well as spiking induced by synaptic input (42). Many cerebellar ataxias indeed show alteration of Ca^2+^ homeostasis as pathogenetic converging mechanism (43, 44). Ca^2+^ imaging experiments disclosed higher cytosolic Ca^2+^-peak responses upon KCl stimulation in *Sacs*^–/–^ PCs compared with WT. Deregulated Ca^2+^ homeostasis at synapses in the absence of sacsin likely results from ineffective Ca^2+^ buffering due to deprivation of mitochondria and ER, but also from reduced levels of ATP in these sites that can impact Ca^2+^ ATP-ase functionality. In vivo, we detected a specific increase of phosphorylation state of CaMKIIβ in *Sacs^–/–^* cerebellum and downregulation of many Ca^2+^ related proteins, including Ca^2+^ ATP-ases, Ca^2+^ binding proteins, and IP3R1.

We also found a striking upregulation of mRNAs typical of the phagocytic microglia and of reactive astrocytes, indicating a neuroinflammatory process accompanying PC degeneration. These findings were complemented by immunofluorescence analyses, showing typical changes in morphology of these glial cells during the neurodegeneration (45, 46). There is growing evidence that microglia may contribute to cerebellar vulnerability in ataxias (47); however, further studies are needed to dissect the role of neuroinflammation as a potential modifier of disease progression in ARSACS.

Deregulated Ca^2+^ homeostasis in *Sacs^–/–^* PCs and cerebellum provided the rationale to test Ceftriaxone efficacy in the ARSACS mouse model. It is indeed well documented that this drug limits glutamatergic stimulation of neurons, reducing Ca^2+^ influx postsynaptically (32), although some papers propose its neuroprotective action through increased antioxidant response and attenuated neuroinflammation (32). We decided to administer Ceftriaxone i.p. at a regimen already effectively proven to target cerebellum (18, 34). Drug treatment resulted successful in symptomatic *Sacs*^–/–^ mice, as it delayed PC loss and attenuated reactive astrogliosis, with a clear arrest of motor impairment progression. The beneficial effect of Ceftriaxone was likely due to its action on Ca^2+^ levels, as shown by a normalized phosphorylation state of CaMKIIβ and by the restoration of IP3R1, calbindin, and parvalbumin levels in the cerebellum of treated *Sacs*^–/–^ mice. Ceftriaxone was previously shown to modulate the transcription of GLT1 through NF-κB (48). Our in silico analysis highlighted NF-κB putative binding sites in the promoter region of *Itpr1*, *Calb1*, and *Pvalb*, suggesting that a similar mechanism could account for the increased levels of these proteins upon Ceftriaxone treatment (Supplemental Figure 7E).

Encouraging results were obtained also with the presymptomatic Ceftriaxone treatment (starting at 1 month of age), where we tested the possibility to prevent or delay ARSACS disease progression. The outcomes obtained seem not to be significantly different compared with postsymptomatic treatment, suggesting that they both delay PC loss and motor defect. Two possible explanations could be that Ceftriaxone did not target the earliest upstream events of ARSACS pathogenesis (i.e., npNFH accumulation and/or others that are still undefined), and/or that administration at 1 month of age may already be too late to start the treatment, as the molecular pathomechanisms are already activated. In fact, although few papers showed that Ceftriaxone is able to resolve protein aggregates (49, 50), it did not rescue the npNFH accumulation in *Sacs*^–/–^ mice.

ARSACS is the second most common form of recessive ataxia worldwide, and no disease-modifying treatment is available for this disabling disorder. Our data on Ceftriaxone efficacy in the ARSACS mouse model offer the first therapeutic perspective to our knowledge for patients with ARSACS in a close-to-human model. Although a clinical trial with Ceftriaxone failed in a cohort of ALS because of the toxic adverse reaction (it was administered via a central venous catheter, chronically at the dose of 4 g/day up to 30 months, and most patients assumed Riluzole at the same time; ref. 51), our data show that the chronic use of Ceftriaxone may be not necessary in a clinical trial in ARSACS. Therefore, a marked reduction of the dosage combined with a pulsed treatment could drastically reduce toxicity. Ceftriaxone may be also administered intramuscularly or s.c., as employed in a clinical trial for Parkinson’s disease, where they are administering Ceftriaxone by intramuscular injection at 1 g per day for Day 1, 3, and 5 per cycle on a 2-weekly cycle (https://clinicaltrials.gov/ct2/show/NCT03413384?term=ceftriaxone&draw=2&rank=3).

The efficacy of Ceftriaxone at postsymptomatic stages in the *Sacs^–/–^* mouse model encourages a future translation in clinics, as most patients with ARSACS are diagnosed only after the onset of gait abnormalities. Many additional studies are of course needed to further advance Ceftriaxone treatments for ARSACS toward clinical application, especially in terms of dosage, route of administration, duration of treatment, toxicity, and identification of noninvasive biomarkers that could help monitoring drug efficacy.

## Methods

### Immunofluorescence in primary PC cultures and cerebellar slices.

Primary cerebellar cultures were derived as previously described. These are mixed cerebellar cultures containing PCs, astrocytes, and other neurons, as required for PC survival and maturation (18). For immunofluorescence, fixed cells were incubated with the following primary antibodies: calbindin 28 kDa (CB300; Swant Inc.), calbindin (214011; Synaptic System GmbH), OxPhos complex IV subunit I (459600; Invitrogen), calreticulin (C4606; Sigma-Aldrich), and npNFH (SMI32, 801701; Calbiochem). Secondary antibodies conjugated with Alexa Fluor 488 and Alexa Fluor 546 (Invitrogen) were used.

Mice at 5 months of age were sacrificed in the presence of anesthesia (2,2,2-Tribromoethanol; Sigma-Aldrich). Transcardially perfusion was performed and then the brain was isolated. Tissues were fixed in 4% paraformaldehyde (2 hours, 4°C) and then dehydrated in 30% sucrose solution (overnight, 4°C); finally, they were included in OCT solution (Bio-Optica Milano). Cryostat sagittal slices were cut at the thickness of 20 μm and conserved at –80°C. Immunofluorescence was performed as described for fixed primary PCs. For plectin and NFH, images were taken at FluoVIEW FV3000RS Confocal (Olympus) at 63***×*** magnification and analyzed with Fiji-ImageJ software (NIH; https://imagej.net/Fiji). Antibodies used for immunostaining: plectin (sc-33649; Santa Cruz Biotechnology Inc.) and total NFH (ab1989; MilliporeSigma). For GFAP (Z0334; Dako, Agilent) and Iba1 (019-19741; Wako, Fujifilm), samples were imaged using DeltaVision Ultra (GE Healthcare) equipped with a 40***×***/NA0.8 objective lens (Olympus). For multicolor imaging, *Z* stacks of individual channels were sequentially acquired, after optimization of imaging parameters such as illumination parameters and exposure time. For larger fields of view, the samples were scanned at lateral steps of 349 μm (i.e., with 10% overlap), and the collected images were computationally stitched as tile mosaic images using the grid/collection stitching plugin provided by the software package SoftWoRx, which is provided by the microscope’s manufacturer. During the process of figure assembling to create panels, original images (at the resolution of 0,167 μm/pixel) were processed with Fiji software and resized by scaling the pixels by interpolation.

### Volumetric analysis of primary PCs.

Stacks of consecutive confocal images of immunofluorescence performed on primary PCs were taken at 0.1 μm intervals using the UltraVIEW Confocal Microscope (PerkinElmer). Analyses of soma, dendrite, mitochondria, and ER volume were performed using Volocity 3D Image Analysis Software (version 5.5.1, PerkinElmer). For mitochondrial and ER volume evaluation, a region of interest (ROI) was drawn to cover the profile of each PC (or dendrites only). A threshold for red signal (mitochondria and ER) and green signal (PCs) was set to exclude the background. We considered mitochondria/ER belonging to PCs those with red signal exclusively intersecting the green signal.

### Mitochondrial live imaging in primary cerebellar cultures.

Primary PC cultures were obtained as described above. At DIV0, prior to plating, cells were infected with a lentivirus expressing mtDsRed2 (Clontech) at 1:200 concentration from 4.67 ***×*** 10^8^ U/mL. Cells were plated on bottom-glass culture dishes (MatTek Corporation). At DIV10, real-time movies of mitochondria in PC-dendrites were acquired on a DeltaVision Ultra (GE Healthcare) microscope enclosed with a temperature and CO_2_ incubation chamber. Images were acquired with a 60***×***/NA1.4 objective lens (Evident) every minute for 30 minutes. Images were first deconvolved with Huygens Professional version 19.04 (Scientific Volume Imaging, Netherlands, http://svi.nl), and mitochondria tracking was performed with Arivis Vision4D (Version 3.1.3, arivis AG Rostock). Before analysis, images were cropped and rotated to orientate the dendrite horizontally and keep the soma always on the left side of the image. A manual track tool was used to create a track interactively in the viewer. Output measurements were then exported in excel files for each track and analyzed for statistical significance. Kymographs were obtained with KimographClear plugin for Fiji (52), from time series of maximum projections, according to user manual, where forward and backward motion correspond to anterograde and retrograde mitochondrial movement, respectively.

### EM analyses.

EM analyses were done in collaboration with the Unit of Neuropathology of San Raffaele Institute as previously described (18) (Supplemental Methods).

### In vivo mitochondrial ATP assay.

To measure mitochondrial ATP production in cerebellum, we isolated fresh mitochondria and applied the same experimental procedure as described in ref. 53 (Supplemental Methods).

### Measurement of ΔΨ_mito_.

ΔΨ_mito_ was measured using TMRM (Invitrogen) as previously described (18) (Supplemental Methods). Images were analyzed using Fiji software.

### COX-SDH enzymatic assay.

In situ activity staining were performed on cryostat sagittal slices according to manufacturer’s instructions (Bio-Optica Milano). Images were acquired using Axio Imager.M2 (Zeiss) and analyzed using Fiji software.

### IP.

SH-SY5Y cells or cerebellum were collected and freshly lysed in lysis buffer (5 mM EDTA [pH 8.0], Triton X-100 0.1% in PBS-1***×*** and protease inhibitor cocktail [PIC]; Sigma-Aldrich) with a Dounce homogenizer. Total homogenate was centrifuged at 8,000*g* for 10 minutes at 4°C. After a preclearing step, the IP antibody was bound to magnetic Dynabeads A or G (Thermo Fisher Scientific) and the Dynabeads-antibody complex was incubated with the precleared lysate overnight at 4°C on a wheel. After washes in lysis buffer, the antigen was eluted in Urea 8M Tris-HCl (pH 8) on a rotation for 30 minutes for liquid chromatography–MS/MS (LC-MS/MS) and/or WB or in Laemmli buffer following a 10-minute rotation, along with incubation at 100°C for 5 minutes, for WB.

For sacsin IPs, anti-sacsin antibody (181190; Abcam) was used; for plectin IP, anti-plectin (ab32528; Abcam) antibody was used; for NFL IP, anti-NFL antibody (8A1, sc-20012; Santa Cruz Biotechnology Inc.) was used. Anti-myosin (LF-18 M4812; Sigma-Aldrich), anti-plectin (ab32528; Abcam), and anti-NFL (8A1, sc-33649; Santa Cruz Biotechnology Inc.) antibodies were used for immunoblotting. Mouse IgG1 (R&D Systems) or rabbit IgG (Sigma-Aldrich, Merck KGaA) were used as isotype controls.

Immunoprecipitated eluates were sent to LC-MS/MS and/or loaded on SDS-PAGE for WB analysis. Enrichment and network analyses of proteins identified as sacsin interactors were performed with stringApp for Cytoscape 3.8.

For the co-IP between NFL and sacsin, an additional step was adopted from the protocol previously developed by Rao et al. to uncover the interaction between NFL and myosin Va (54). Briefly, SH-SY5Y cells were lysed in Triton X-100 1%, Tris-HCl (pH 6.8) 50 mM, NaCl 200 mM, glycerol 20%, and EDTA 1 mM with Douncer homogenizer; a Triton-insoluble pellet was resuspended in Tris-buffered saline, and SDS 10% and NF cytoskeletal insoluble fraction was extracted by 1:4 dilution in NF extraction buffer (Tris-HCl [pH 7.4] 60 mM, NaCl 190 mM, EDTA 6 mM, Triton-X100 1.25%), prior to incubation with Dynabead-antibody complexes.

### Tissue lysis and antibodies for WB.

For WBs, soluble fractions were obtained from tissues or cells by lysis in 100 mM Tris-HCl (pH 7.4), 1 mM EDTA (pH 8), 1% Triton X-100, and 150 mM NaCl supplemented with PIC (Sigma-Aldrich) and phosphatase inhibitor cocktail (Merck KGaA) using a Dounce homogenizer and incubated for 30 minutes on ice. Cell debris were discarded by centrifugation at 8,000*g* for 10 minutes at 4°C. To obtain insoluble fractions, tissues were homogenized in high ionic strength buffer (0.05M MOPS [pH 6.8], 1% Triton X-100, and 0.6M KCl) and then centrifuged at 15,000*g* for 5 minutes at room temperature. The triton insoluble pellet was treated with 0.5 μg/mL DNase I in 10 mM MgCl_2_ buffer for 30 minutes at 37°C. After washing, the pellet was solubilized in urea buffer (8M urea in 0.1M MOPS).

Commercially available antibodies were used for the detection of CaMKIIβ (sc-376828), pCaMKIIβ (sc-12886), and PCP2 (sc-137064) (all from Santa Cruz Biotechnology Inc.); npNFH (SMI32, 801701; BioLegend); plectin (ab32528; Abcam); PSD95 (ab2723; Abcam); sacsin (ab181190; Abcam); IP3R1 (NB120-5908; Novus Biologicals); calbindin1 (214011; Synaptic System GmbH); GFAP (Z0334; Dako, Agilent); calnexin (C4731; Sigma-Aldrich); and myosin Va (LF-18, M4812; Sigma-Aldrich). Secondary antibodies included horseradish peroxidase–conjugated (HRP-conjugated) anti-mouse and anti-rabbit IgG (Amersham Bioscience).

### Ca^2+^ imaging.

Ca**^2+^** peaks were assayed with Calbryte 520 (AAT Bioquest). Cell loading was performed at 37°C (5 μM, 30 minutes) in HBSS 1***×*** buffer. Images were acquired on a widefield Zeiss Axio-Observer.Z1 microscope equipped with a 20***×*** objective lens (Carl Zeiss Microscopy). The evoked Ca^2+^-response (fold change, ΔF/F_0_) was calculated as mean values within ROIs drawn in neuronal soma (Fiji software).

### qPCR.

cDNA was generated using SuperScript IV Reverse Transcriptase kit (Invitrogen) and processed by qPCR using the SYBR green chemistry (Light cycler 480, SYBR green I master; Roche).

To perform qPCR, we used the following primers (5′–3′): *Itpr1*: forward (FW), GGCTACAGGGCATTACTTGG; reverse (REV), GATGGAGGAGATGTCGTTGC; *Calb1*: FW, AGTTGGCTCACGTCTTACCC; REV, CTCTGTCAGTTCCAGCTTTCC; *Casq2*: FW, CACGTACGATGGGAAAGACC; REV, ATCCCAGCCTCTTAGCAAGC; *Car8*: FW, CTTGCAGCGAAGGAGTTACC; REV, GGTAGGTCGGAAATTGTCTC; *Gfap*: FW, GTGGAGAGGGACAACTTTGC; REV, CTCCTCCAGCGATTCAACC; and *Hprt1*: FW, ACATTGTGGCCCTCTGTGTG; REV, TTATGTCCCCCGTTGACTGA.

### LFQ proteomics analysis.

Mouse cerebellum (5 months of age) was lysed in 8M urea, 100 mM Tris-HCl (pH 8), and PIC. Samples were processed by LFQ-MS/MS in collaboration with Cogentech proteomics facility (IFOM). To determine the significance of the differential proteins was used the cut-off determined by FDR < 0.05 (*n* = 3 WT and 3 *Sacs^–/–^* mice, each in technical replicate). The significant deregulated proteins obtained in this way were submitted to g:Profiler Enrichment analysis (https://biit.cs.ut.ee/gprofiler/gost).

### Transcriptomics analysis.

Standard RNA-Seq analysis was performed on total RNA extracted from cerebellum with RNeasy kit (Qiagen) (*n* = 5 WT and 5 *Sacs^–/–^* mice). Libraries were prepared using True-Seq stranded mRNA for mRNA-Seq (Illumina). Sequencing was performed on a NextSeq 500 machine (Illumina) obtaining 30 million single-end reads per sample on average. Only genes with a counts per million (CPM) value higher than 1 in at least 3 samples were retained. Gene expression read counts were exported and analyzed in R environment (v. 3.6.2) to identify differentially expressed genes (DEGs). The DEG analysis was performed with the package DESeq2 available in Bioconductor comparing different experimental groups. To determine the significance of the differential genes was used the cut-off determined by FDR filter < 0.1 (adjusted *P* value). The significant deregulated genes obtained with this algorithm were submitted to g:Profiler Enrichment analysis.

### Animals and Ceftriaxone treatments.

*Sacs^–/–^* and WT littermates were obtained by breeding *Sacs^+/–^* male and female mice (C57BL/6). Ceftriaxone (Fidia Farmaceutici) was administered monthly by daily i.p injection at the dose of 200 mg/kg body weight for 5 consecutive days as previously described (18).

### Behavioral tests.

BW test to assess motor balance was performed for 3 consecutive days (in each day the mice performed 3 trials on the beam, 7 mm ***×*** 90 cm suspended 40 cm above bedding), after 2 days of training. The number of hindfoot missteps and the time required to cross the beam (latency) was evaluated, as previously described (55). The mean of all the trials was scored. Animal behavioral testing was performed by investigators blinded to the group of the mice; the analysis was performed by 2 independent investigators analyzing videorecording of the motor tests.

### Histological analyses.

Tissues were fixed after perfusion in 4% paraformaldehyde and 2.5M glutaraldehyde in 0.12M cacodylate buffer solution. Semithin sections (1 μm) of cerebellum were cut and stained with toluidine blue. Images of anterior lobules of cerebellum were acquired on Olympus BX51 microscope equipped with a 20***×*** objective lens (Leica Microsystems).

### Statistics.

For statistical evaluation of phenotypes (imaging and WB) in *Sacs^–/–^* cells or mice compared with WT controls, we performed unpaired Student’s *t* test, 2-tailed (applying Welch’s correction). For behavioral tests, power analysis for sample size estimation was performed using G*Power (v. 3.1) software based on previous data to achieve power set at 80% and significance level at *P* < 0.05. Regarding pharmacological treatment with Ceftriaxone, for statistical comparisons, we applied 2-way ANOVA and Tukey’s multiple-comparison test (GraphPad Prism software, https://www.graphpad.com/scientific-software/prism/).

### Study approval.

The preclinical trials with Ceftriaxone were approved by and performed in accordance with experimental protocols approved by the IACUC of San Raffaele Scientific Institute.

### Data availability.

Raw RNA-Seq data have been uploaded in GEO repository (GSE200876), and proteomics data have been uploaded in PRIDE repository (PXD033385) (GEO, https://www.ncbi.nlm.nih.gov/geo/; PRIDE, https://www.ebi.ac.uk/pride/).

## Author contributions

FM, ADB, and FL conceptualized the study. FM, ADB, and FL designed the study methodology. ADB, FL, DDR, ES, PP, and AB performed experiments. FM, ADB, FL, DDR, ES, and AQ analyzed data. FM acquired funds. AB, AQ, and BB provided expertise and feedback. FM and ADB wrote the manuscript. All authors reviewed the manuscript.

## Supplementary Material

Supplemental data

Supplemental table 1

Supplemental table 2

Supplemental table 3

Supplemental table 4

Supplemental video 1

Supplemental video 2

Supplemental video 3

Supplemental video 4

Supplemental video 5

Supporting data values

## Figures and Tables

**Table 1:**
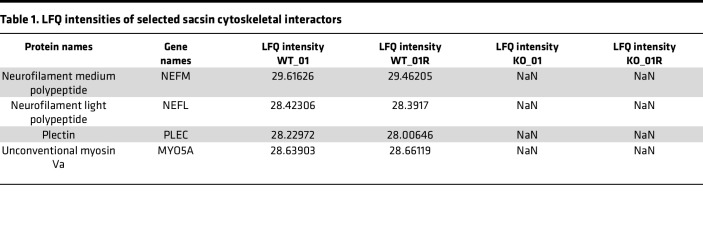
LFQ intensities of selected sacsin cytoskeletal interactors

## Version 1 05/09/2023

In-Press Preview

## Version 2 06/22/2023

Electronic publication
