# Supplementary material for: Restoring calcium homeostasis in Purkinje cells arrests neurodegeneration and neuroinflammation in the ARSACS mouse model
Source: JCI Insight. 2023 Jun 22;8(12):e163576. doi: 10.1172/jci.insight.163576 (PMC10371240; doi:10.1172/jci.insight.163576)
Supplement: Supplemental data [file jciinsight-8-163576-s152.pdf]

Supplemental methods

In vivo mitochondrial ATP assay

Isolated mitochondria were incubated at 37°C for 30 min in a respiratory buffer (0.25 M sucrose, 20 mM MOPS, 1 mM EDTA, 5 mM inorganic phosphate, 0.1% BSA fatty acid free, and 1mM ADP, pH 7.4) containing specific substrates of the respiratory chain complexes. By providing pyruvate/malate (5 and 1 mM, respectively) and glutamate/ malate (5 and 1 mM, respectively), we stimulated ATP synthesis dependent on complexes I, II, III, IV, and V. ATP production was measured by luminometric assay: ATP concentration was determined with the luciferin-luciferase method. The assay solution was prepared as follows: 250 mM glycylglycine, 2 mM EGTA, 2 mM MgCl2, 0.4 g/L BSA fatty acid free, 7.5 mM DTT, 15 µM luciferin, and 10 µg/ml luciferase. Isolated mitochondria were lysed with the ATP lysis buffer (0.2 M NaOH and 0.5 mM EDTA), and an aliquot of the obtained extract was diluted with the ATP dilution buffer (0.1 M NaOH and 0.5 mM EDTA). Twenty microliters of this mixture were added to 100 µl of the assay solution, and the ATP content was measured by luminometer. Results are expressed as nanomoles of ATP per milligram of protein.

Measurement of $\Delta\Psi_{mito}$

$\Delta\Psi_{mito}$ was measured using the potentiometric dye TMRM (Invitrogen). Primary PCs at 15 DIV were incubated with 50 nM TMRM, 2 mM CsH (Vinci-Biochem), and 2 mg/ml Hoechst 33342 (Invitrogen) in phenol red–free HBSS 1× for 30 minutes at 37°C. Imaging of TMRM fluorescence was performed using an Axio Observer.Z1 inverted microscope (Zeiss). Data represent the average of 4 images acquired every 15 seconds. FCCP (1 µM) was added at the end of acquisition. Images were analyzed using Fiji software.

Antibodies for WB

Commercially available antibodies were used for the detection PSD95 (ab2723), PGC1α (ab191838; Abcam), vimentin (ab92547; Abcam), spectrin (MAB1622; Millipore, Merck

KGaA), calbindin1 (Synaptic System GmbH), GFAP (Dako, Agilent), calreticulin (C4606; Sigma-Aldrich, Merck KGaA), GAPDH (sc-32233; Santa Cruz), YME1L1 (11510-1-A-P; Proteintech Inc.), Parvalbumin (PV27; Swant Inc.) and AFG3L2 (homemade). Secondary antibodies included Horseradish Peroxidase (HRP)-conjugated anti-mouse and anti-rabbit IgG (Amersham Bioscience).

Immunofluorescence in SH-SY5Y cells and primary PC cultures

SACS$^{-/-}$ SH-SY5Y clones were previously generated by CRISPR/Cas9 genome editing [19] and differentiated by retinoic acid (Sigma-Aldrich, Merck KGaA) 10 uM (7days) and BDNF (Sigma-Aldrich, Merck KGaA) 50ng/mL (7 days).

For immunofluorescence, fixed cells were incubated with the following primary antibodies: calbindin 28 kDa (CB300; Swant Inc.), calbindin (244011; Synaptic System GmbH), npNFH (SMI32, 801701; Biolegend), GFAP (Z0334; Dako, Agilent), Phalloidin (A22287 Invitrogen), α-tubulin (236-10501; Invitrogen), NFH (ab1989; Millipore), and plectin (sc-33649; Santa Cruz) and vimentin (ab92547; Abcam). Secondary antibodies conjugated with Alexa Fluor 488 and Alexa Fluor 546 (Invitrogen) were used.

Immunofluorescence of sacsin and its interactors was performed in primary PCs as described above, with the addition of following steps for sacsin detection: after cell fixation, antigen retrieval was performed incubating cells in Tris 10mM, EDTA 1mM, Tween-20 0.05% at pH 9.0 for 30’ at 70-90°C; then cells were washed with DPBS1X and pre-blocked for 20’ with 50mM glycine in DPBS1X; Bovine Serum Albumin 0.2% was added in blocking buffer. Antibodies used for IF: sacsin (181190; Abcam), plectin (sc-33649; Santa Cruz) and npNFH (SMI32, 801701; Biolegend); PCs were marked with chicken-IgY anti-calbindin (A85359; Antibodies.com). In cryostat sections, PCs were marked with chicken-IgY anti-calbindin (NBP2-50028SS; Novus Bio, Bio-techne) .

qRT-PCR

To perform qRT-PCR were used the following primers (5'>3'):

Pgc1α FW - GAATCAAGCCACTACAGACACCG

REV - CATCCCTCTTGAGCCTTTCGTG

Nrf1 FW - GGCAACAGTAGCCACATTGGCT REV - GTCTGGATGGTCATTTCACCGC

Nrf2 FW - ACTTGGAGTTGCCACCGCCA REV - TGTGCTGGGCCGGCTGAATT

Hprt1 FW - ACATTGTGGCCCTCTGTGTG REV - TTATGTCCCCCGTTGACTGA

Synaptosome purification from mouse cerebellum

Mouse cerebellum was homogenized in sucrose buffer (0,32 M sucrose, 4 mM HEPES pH 7.4 and protease inhibitors). Samples were then centrifuged at 1000 g 10' at 4°C to pellet debris and nuclei. Supernatant was centrifuged at 10000 g 15' at 4°C to obtain crude synaptosomal fractions. The pellets were washed once in the same buffer and centrifuged again at the same speed. Pellets underwent an osmotic shock by resuspending in aqueous buffer (4 mM HEPES pH 7.4 and protease inhibitors) and rotating 30' at 4°C. They were then centrifuged at 25000 g 20' at 4°C obtaining the synaptosomes.

EM image analysis

EM were done in collaboration with the Unit of Neuropathology of San Raffaele Institute. Cerebellum was incubated in glutaraldehyde fixative to perform standard EM experiments. Several cells were evaluated in a single sample for a total 3 *Sacs*$^{-/-}$ versus 3 wild-type tissues. Morphometric analysis of mitochondria in EM images was performed using the freehand line selection tool of ImageJ software to calculate mitochondria area and perimeter, major and minor axis, circularity, and roundness. Circularity is 4π*area/perimeter^2. A value of 1.0 indicates a perfect circle. While roundness is the inverse of the aspect ratio (major axis/minor axis) and is calculated as follows: 4 * area/(π * major_axis^2).

Haematological and toxicological analyses

To assess the health state of mice treated with Ceftriaxone, just before the sacrifice, as much blood as possible was collected via eyeball. The blood was then analysed to measure haematological values and clinical chemistry parameters to evaluate kidney and liver functionality by following standard mouse clinic protocols.

Supplemental figures with legends

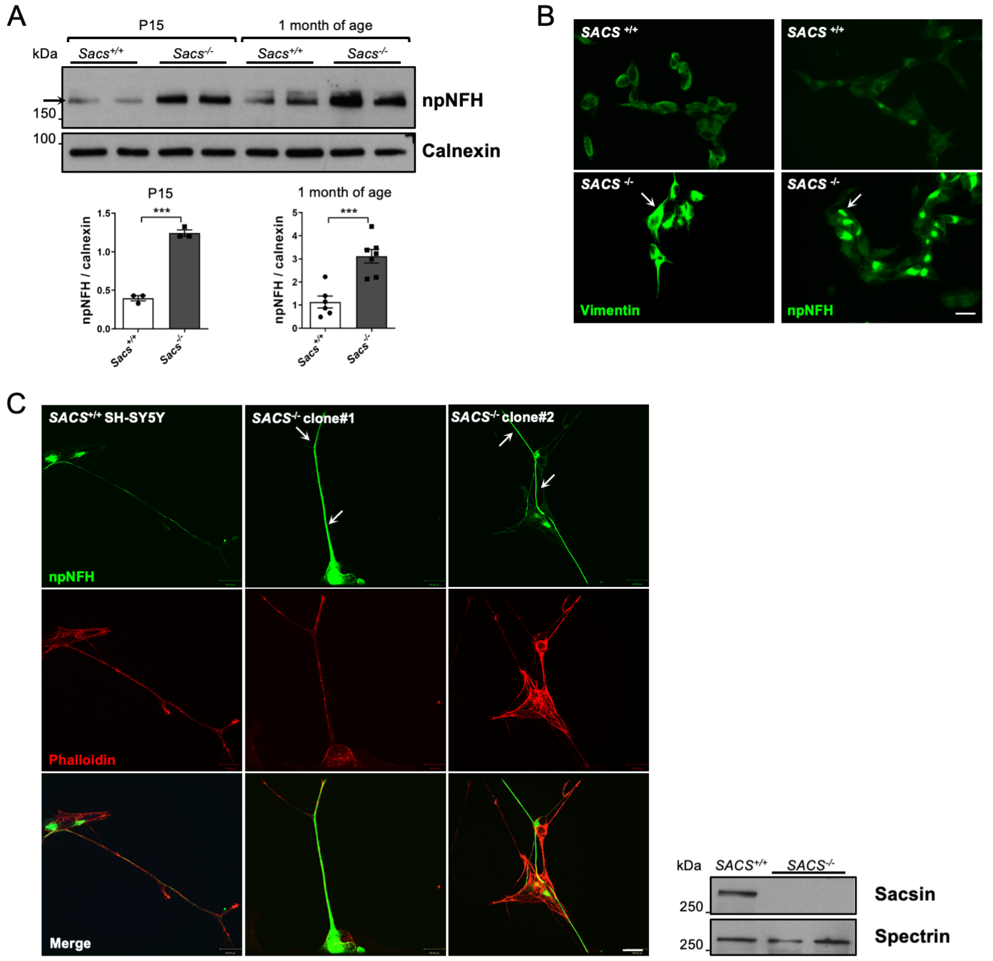

Supplemental figure 1. (**A**) WB analysis showing levels of npNFH (lower band as indicated by the arrow) in *Sacs*$^{-/-}$ and wild-type control cerebellum at P15 and 1 month of age with relative quantitation (normalized to calnexin, which was not significantly altered in LFQ proteomics, see Supplemental Table 2). Bars represent mean±SEM; n= at least 3; Welch's t-test: ***P<0,001; (**B**) Representative images (63X) of immunofluorescence analysis showing vimentin and npNFH accumulation in undifferentiated *SACS*$^{-/-}$ SH-SY5Y cells compared with *SACS*$^{+/+}$ controls. Arrows indicate vimentin and npNFH accumulation. Scale bar=13 µm; (**C**) Representative images (63X) of immunofluorescence analysis showing npNFH accumulation in neuron-like differentiated *SACS*$^{-/-}$ SH-SY5Y cells compared with *SACS*$^{+/+}$ controls. Scale bar=13µm. WB to check sacsin levels in the clones. Arrows indicate npNFH accumulation.

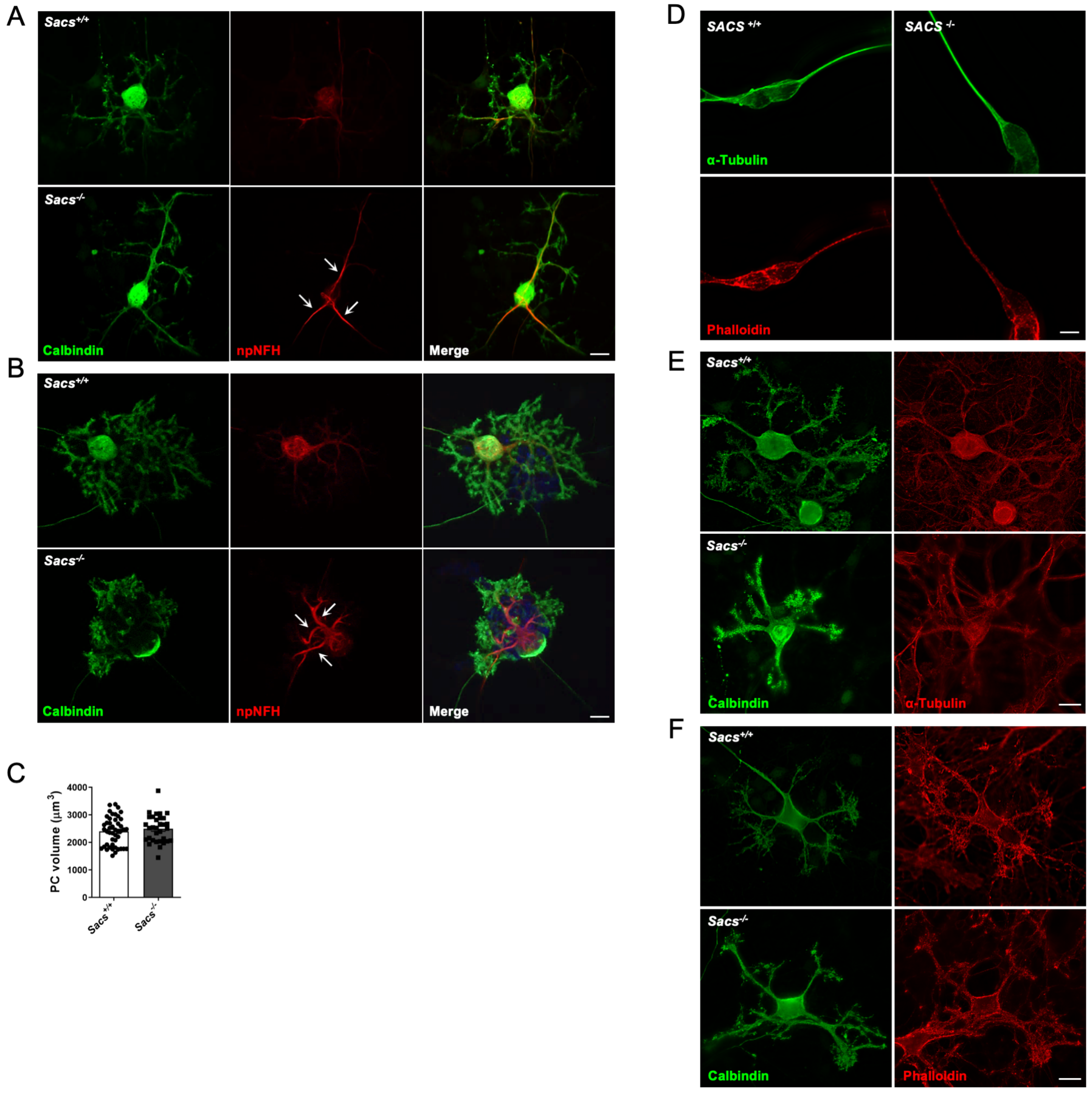

Supplemental figure 2. (**A-B**) Representative confocal images (63X) of DIV10 (A) and DIV15 (B) *Sacs*$^{-/-}$ and wild-type PCs stained in green with calbindin, while in red with npNFH. Arrows highlight npNFH accumulation. Scale bar=13µm; (**C**) Graph represents volumetric quantification of total PC volume (µm^3). Bars represent mean±SEM; n=at least 32 from at least 10 independent experiments; Welch's t-test: ns; (**D**) Representative images (63X) of immunofluorescence analysis showing α-tubulin (in green) and Phalloidin (in red) in neuron-like differentiated *SACS*$^{-/-}$ SH-SY5Y cells compared with *SACS*$^{+/+}$ controls. Scale bar=13µm; (**E-F**) Representative confocal images (63X) of DIV15 *Sacs*$^{-/-}$ and wild-type PCs stained in green with calbindin, while in red α-tubulin (E) and Phalloidin (F). Scale bar=15µm.

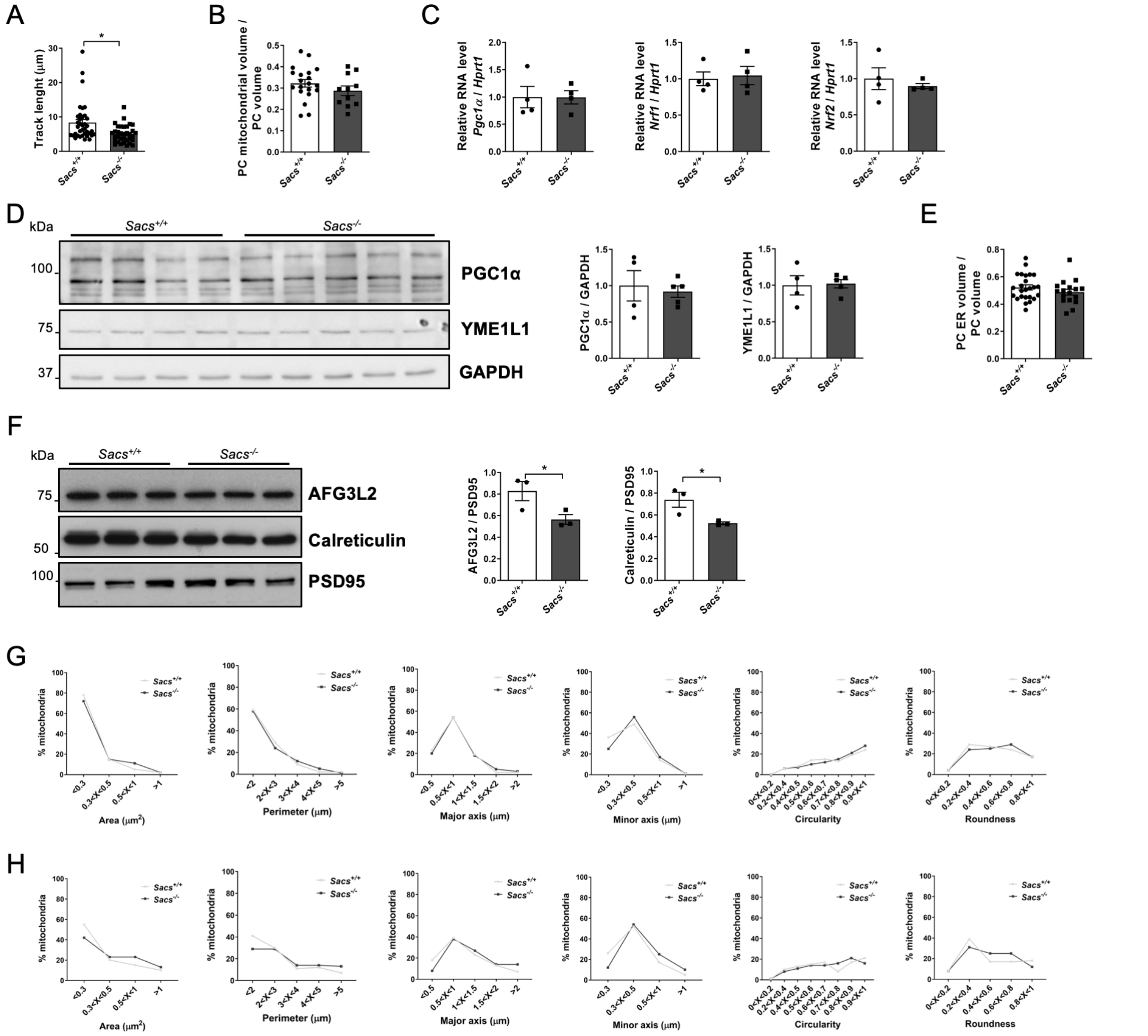

Supplemental figure 3. (**A**) Graph represents track length of each mitochondrion involved in the analysis (referring to Fig. 1B). Bars represent mean±SEM; n=at least 34 from at least 3 independent experiments; (**B**) Graph represents volumetric quantification of total PC mitochondrial volume (normalized to total PC volume). Bars represent mean±SEM; n=at least 11 from at least 5 independent experiments; Welch's t-test: ns; (**C**) qRT-PCR showing levels of *Pgc1α*, *Nrf1* and *Nrf2* mRNA (relative to *Hprt1* mRNA) in $Sacs^{-/-}$ and wild-type control cerebellum at 5 months of age. Bars represent mean±SEM; *n*=4; Welch's t-test: ns; (**D**) WB analysis showing levels of PGC1α (all the bands) and YME1L1 in $Sacs^{-/-}$ and wild-type control cerebellum at 5 months of age with relative quantitation (normalized to GAPDH). Bars represent mean±SEM; n=at least 4; Welch's t-test: ns; (**E**) Graph represents volumetric quantification of total PC ER volume (normalized to total PC volume). Bars represent mean±SEM; n=at least 17 from at least 6 independent experiments; Welch's t-test: ns; (**F**) WB analysis showing levels of AFG3L2 (mitochondrial marker) and calreticulin (ER marker) in $Sacs^{-/-}$ and wild-type control cerebellar synaptosomes at 5 months of age with relative quantitation (normalized to PSD95). Bars represent mean±SEM; n=3; Welch's t-test: *P <0,05; (**G-H**) Morphometric analysis EM in vivo (G, referring to Fig. 2A) and ex vivo (H, referring to Fig. 2E) mitochondria images indicating frequency distribution of each parameter (area, perimeter, major and minor axis, circularity and roundness).

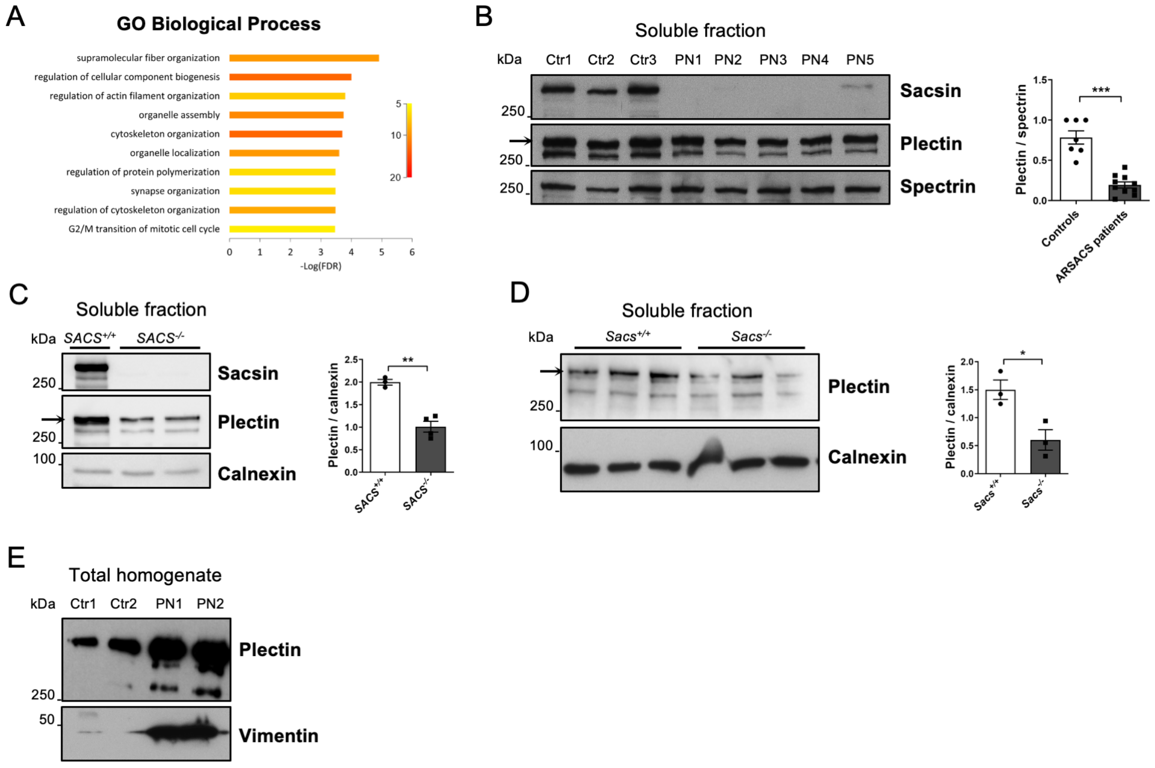

Supplemental figure 4. (**A**) Gene Ontology (Biological Process) STRING enrichment output of sacsin co-immunoprecipated proteins; (**B-C**) WB analysis showing levels of plectin (upper band as indicated by the arrow) in ARSACS patient fibroblasts [19] and controls (B) and in *SACS*$^{-/-}$ and *SACS*$^{+/+}$ SH-SY5Y cells (C) with relative quantitation (normalized to calnexin). Bars represent mean±SEM; n=at least 3; Welch's t-test: ***P*<0,01, ****P* <0,001; (**D**) WB analysis showing levels of plectin (upper band as indicated by the arrow) in soluble fractions of *Sacs*$^{-/-}$ and wild-type cerebellum (normalized to calnexin); Bars represent mean±SEM; n=3; Welch's t-test: **P*<0,05; (**E**) Acrylamide-agarose gel analysis showing levels of plectin and vimentin in ARSACS patient fibroblasts [19] and relative controls.

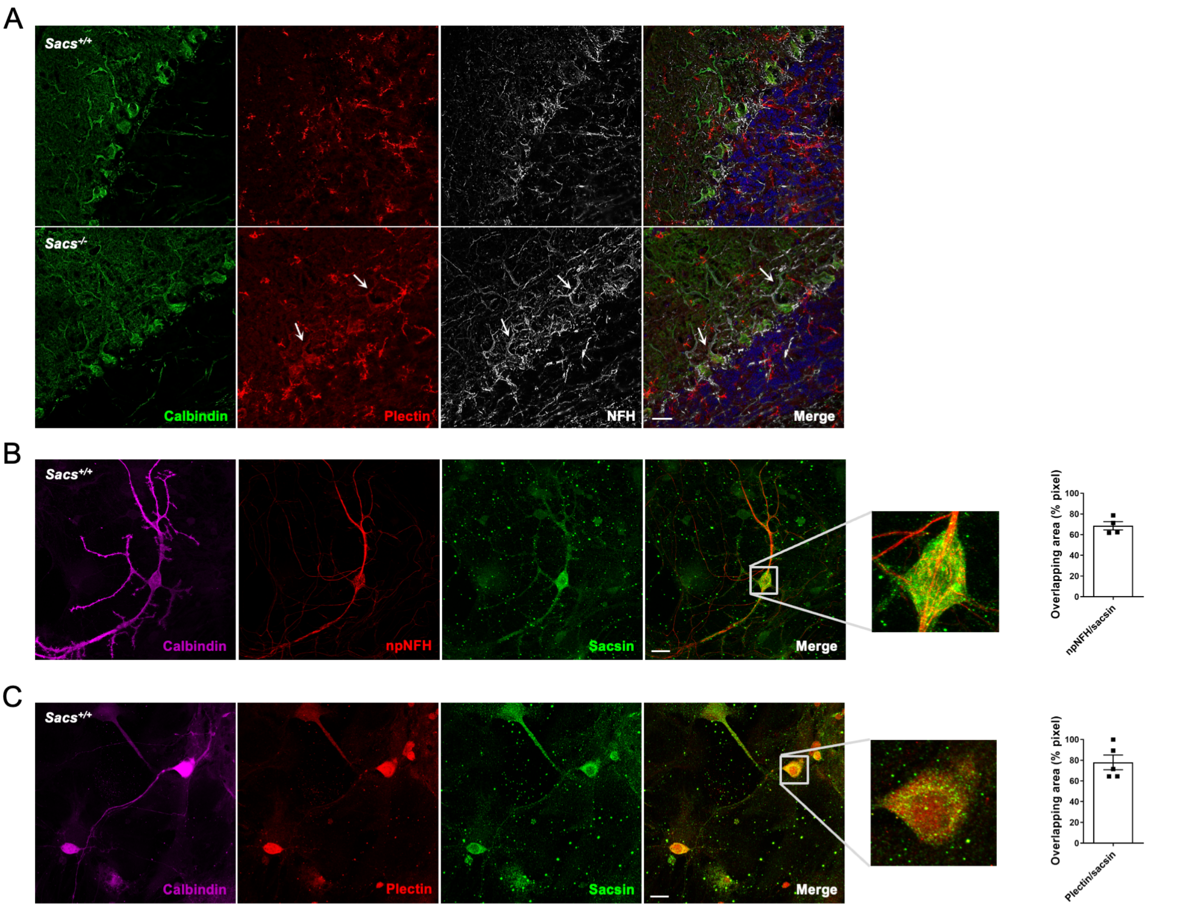

Supplemental figure 5. (**A**) Representative confocal images (60X) of cerebellar slices stained with calbindin (in green), plectin (in red) NFH (in white) showing plectin and NFH accumulation in *Sacs*$^{-/-}$ PC soma and proximal dendrites, absent in wild-type controls. Scale bar=15µm. (**B-C**) Representative confocal images of *Sacs*$^{+/+}$ PCs stained in magenta with calbindin, in red with npNFH (B) or plectin (C) and in green with sacsin underliying high signal co-localization. Graph represents the area of overlapping signals (measured in pixel percentage). Scale bar=20 µm. Bars represent mean±SEM; n=4 from 4 independent experiments.

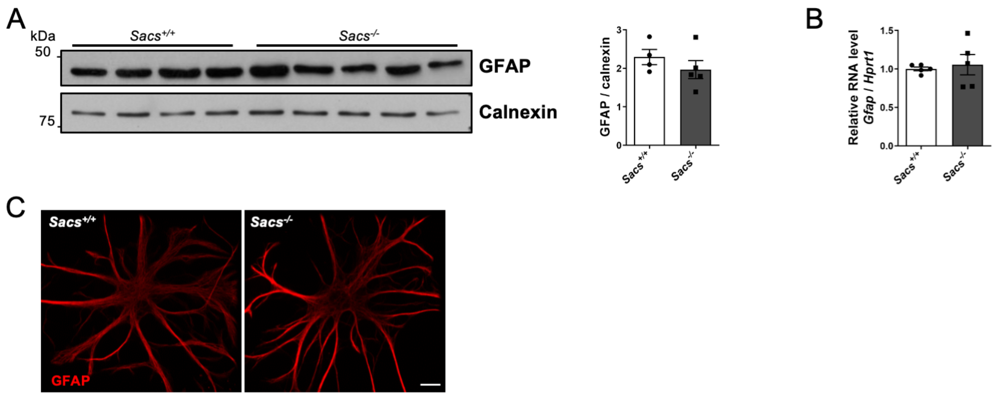

Supplemental figure 6. (**A**) WB analysis showing levels of GFAP in *Sacs*$^{-/-}$ and wild-type control cerebellum at 1 month of age with relative quantitation (normalized to calnexin). Bars represent mean±SEM; n=at least 4; Welch's t-test: ns; (**B**) qRT-PCR showing levels of *Gfap* mRNA (relative to *Hprt1* mRNA) in *Sacs*$^{-/-}$ and wild-type control cerebellum at 1 month of age. Bars represent mean±SEM; n=5; Welch's t-test: ns; (**C**) Representative confocal images (63X) of DIV15 *Sacs*$^{-/-}$ and wild-type astrocytes stained in red. Scale bar=13µm.

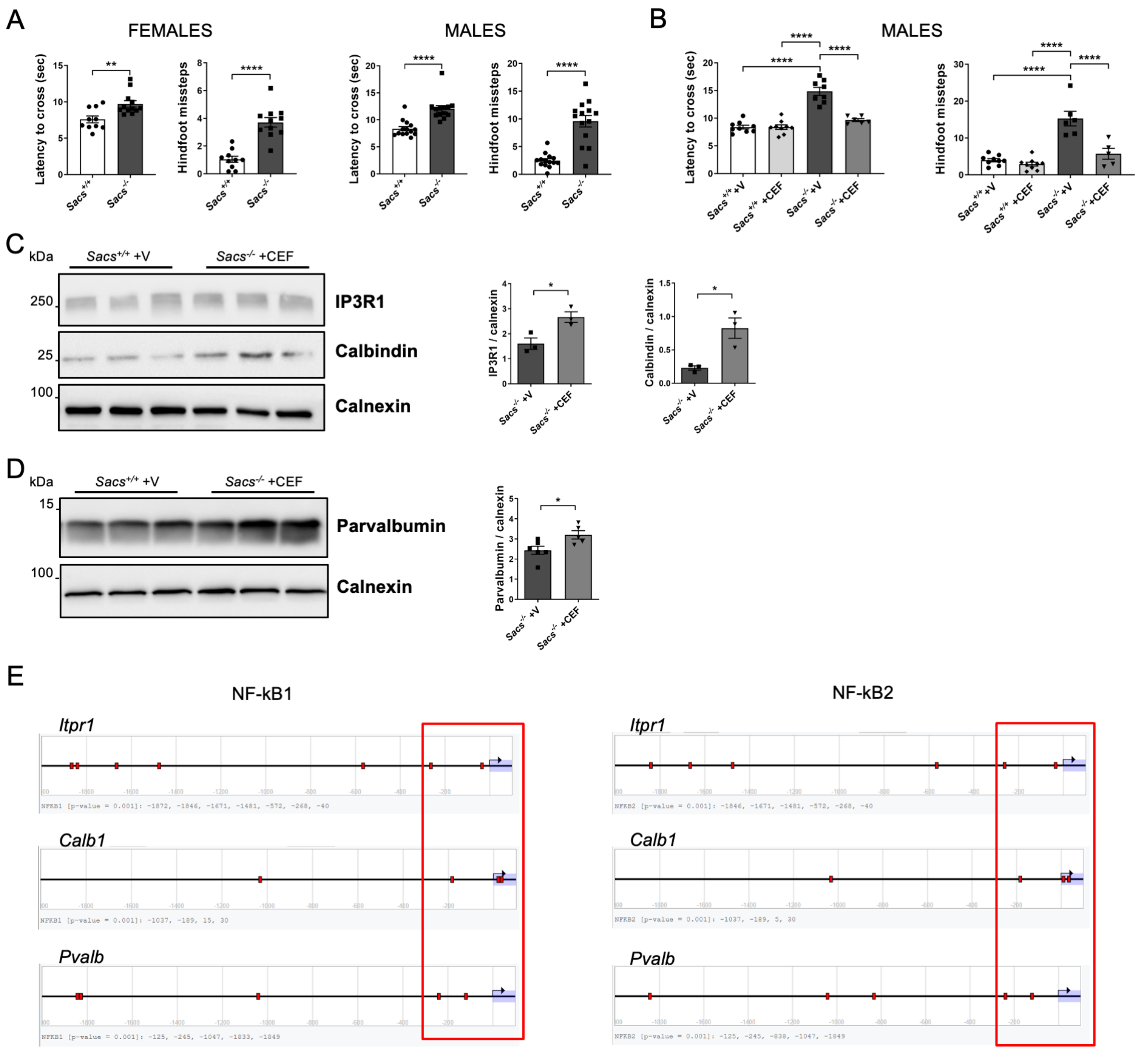

Supplemental figure 7. (**A**) Pre-treatment (5 month of age) motor assessment by BW test readouts of latency time to cross the beam and number of hindfoot missteps both in female and male mice. Bars represent mean±SEM; n= at least 10; Welch's t-test: **p<0,01, ****p<0,0001; (**B**) BW test performance in term of latency time to cross the beam and number of hindfoot missteps at 7 months of age male Ceftriaxone- and vehicle-treated mice. Bars represent mean±SEM; n=at least 6; Two-way ANOVA with Tukey's correction: ****p<0,0001; (**C-D**) WB analysis showing levels of IP3R1 and Calbindin (C) and Parvalbumin (D) in *Sacs*$^{-/-}$ vehicle- and Ceftrixone-treated cerebellum at 7 month of age (post-symptomatic Ceftriaxone treatment) with relative quantitation (normalized to calnexin). Bars represent mean±SEM; n=at least 3; Welch's t-test: *p<0,05; (**E**) *Mus musculus* NF-kappa-B putative binding sites (p-value <0.001). Data of putative binding sites (red hits) were retrieved for *Nfkb1* and *Nfkb2* with EPD search motif tool (Eukaryotic Promoter Database, available at https://epd.epfl.ch/), scanning from -2000 to +100 in respect to TSS, according to JASPAR core 2018 vertebrates library. *Nfkb1* and *Nfkb2* encode respectively for the p105 and p100 DNA-binding subunits of NF-kappa-B transcription factor.

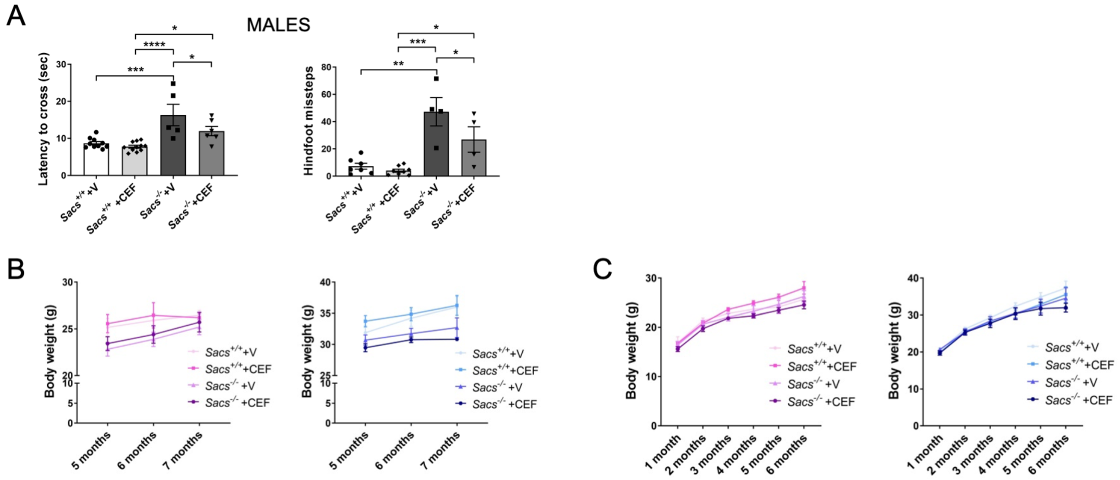

Supplemental figure 8. (**A**) BW test performance in term of latency time to cross the beam and number of hindfoot missteps at 6 months of age male Ceftriaxone- and vehicle-treated mice. Bars represent mean±SEM; n=at least 4; Two-way ANOVA with Tukey's correction: *p<0,05, **p<0,01, ***p<0,001, ****p<0,0001; (**B-C**) Monthly body weight check-up of both *Sacs*$^{-/-}$ and wild-type post-symptomatic (B) and pre-symptomatic (C) Ceftriaxone- and vehicle-treated female/male mice. Dots represent mean±SEM; n=at least 6; Welch's t-test: ns.

Supplemental Movie 1. Representative live-imaging recording of mitochondrial movement in *Sacs*$^{+/+}$ primary PCs.

Supplemental Movie 2. Representative live-imaging recording of mitochondrial movement in *Sacs*$^{-/-}$ primary PCs.

Supplemental Movie 3 . Representative BW test of a wild-type female mouse at 7 months of age (post-symptomatic trial).

Supplemental Movie 4. Representative BW test of a *Sacs*$^{-/-}$ vehicle-treated female mouse at 7 months of age (post-symptomatic trial).

Supplemental Movie 5. Representative BW test of a *Sacs*$^{-/-}$ Ceftriaxone-treated female mouse at 7 months of age (post-symptomatic trial).

Supplemental Table 1. Sacsin interactors in neuron-like wild-type SH-SY5Y cells (*SACS*$^{-/-}$ SH-SY5Y cells used as negative control)

Supplemental Table 2. Dataset of deregulated proteins in *Sacs*$^{-/-}$ cerebellum compared with wild-type at 5 months of age

Supplemental Table 3. Dataset of deregulated genes in *Sacs*$^{-/-}$ cerebellum compared with wild-type at 5 months of age

Supplemental Table 4. Haematological and toxicological analyses in 6 months old mice (pre-symptomatic Ceftriaxone trial)
