# Supplementary material for: Restoring calcium homeostasis in Purkinje cells arrests neurodegeneration and neuroinflammation in the ARSACS mouse model
Source: JCI Insight. 2023 Jun 22;8(12):e163576. doi: 10.1172/jci.insight.163576 (PMC10371240; doi:10.1172/jci.insight.163576)
Supplement: Supplemental table 4 [file jciinsight-8-163576-s156.pdf]

	Sacs$^{+/+}$ +V	*Sacs*$^{+/+}$ +CEF	*Sacs*$^{-/-}$ +V	*Sacs*$^{-/-}$ +CEF	**Physiological range**
	**Mean ± SD**	**Mean ± SD**	**Mean ± SD**	**Mean ± SD**	
**RBC (M/μL)**	11.2 ± 0.2	10.4 ± 0.2	10.8 ± 0.4	10.8 ± 0.4	8,60-11
**HGB (g/dL)**	15.5 ± 0.3	14.7 ± 0.2	15.0 ± 0.6	15.1 ± 0.5	12,5-16
**HCT (%)**	51.8 ± 0.8	48.8 ± 0.3	49.6 ± 1.8	49.6 ± 1.6	39-55
**MCV (fL)**	46.3 ± 1.2	46.9 ± 0.7	46.1 ± 0.2	45.8 ± 0.7	42-48
**MCH (pg)**	13.9 ± 0.2	14.1 ± 0.2	13.9 ± 0.3	13.9 ± 0.3	13,5-15,5
**MCHC (g/dL)**	30.0 ± 0.4	30.1 ± 0.2	30.1 ± 0.7	30.4 ± 0.2	30-34
**PLT (K/μL)**	1332.3 ± 91.7	1266.7 ± 496.4	1288.3 ± 245.8	1197.6 ± 195.5	750-1200
**WBC (K/μL)**	5.4 ± 0.9	4.6 ± 0.6	5.3 ± 1.3	6.8 ± 2.1	3,5-10
**NEUT (%)**	21.2 ± 4.9	16.3 ± 5.7	14.8 ± 6.9	14.1 ± 7.7	10-20
**LYMPH (%)**	76.3 ± 5.3	80.4 ± 5.5	82.7 ± 7.1	83.3 ± 8.8	65-85
**ALB (g/dL)**	3.0 ± 0.1	3.0 ± 0.2	3.1 ± 0.2	3.0 ± 0.2	2,7-3,6
**ALT (U/L)**	46.7 ± 15.6	45.7 ± 10.0	53.5 ± 23.2	43.5 ± 13.5	0-70
**Crea (mg/dL)**	0.3 ± 0.0	0.3 ± 0.0	0.2 ± 0.1	0.3 ± 0.0	0,31-0,40
**Urea (mg/dL)**	36.3 ± 9.3	39.0 ± 11.8	43.5 ± 9.9	50.2 ± 6.9	35-50
